# Supplementary material for: Evaluation of a community-based performance arts programme for people who have experienced stroke in the UK: protocol for the SHAPER-Stroke Odysseys study
Source: BMJ Open. 2022 Mar 11;12(3):e057805. doi: 10.1136/bmjopen-2021-057805 (PMC8919447; doi:10.1136/bmjopen-2021-057805)
Supplement: Author's manuscript [file bmjopen-2021-057805.draft_revisions.pdf]

BMJ Open

BMJ Open is committed to open peer review. As part of this commitment we make the peer review history of every article we publish publicly available.

When an article is published we post the peer reviewers' comments and the authors' responses online. We also post the versions of the paper that were used during peer review. These are the versions that the peer review comments apply to.

The versions of the paper that follow are the versions that were submitted during the peer review process. They are not the versions of record or the final published versions. They should not be cited or distributed as the published version of this manuscript.

BMJ Open is an open access journal and the full, final, typeset and author-corrected version of record of the manuscript is available on our site with no access controls, subscription charges or pay-per-view fees (http://bmjopen.bmj.com).

If you have any questions on BMJ Open's open peer review process please email info.bmjopen@bmj.com

BMJ Open

Evaluation of a community-based performance arts programme for people that have experienced stroke – protocol for the SHAPER-Stroke Odysseys study

Journal:	*BMJ Open*
Manuscript ID	bmjopen-2021-057805
Article Type:	Protocol
Date Submitted by the Author:	28-Sep-2021

Complete List of Authors:	Estevao, Carolina; King's College London, nstitute of Psychiatry, Psychology & Neuroscience<br>Baldellou Lopez, Maria ; King's College London Institute of Psychiatry Psychology and Neuroscience, Centre for Implementation Science<br>Davis, Rachel; Florence Nightingale School of Nursing, James Clerk Maxell Building<br>Jarret, Lucinda; Rosetta Life Head Office<br>Soukup, Tayana ; King's College London, Centre Implementation Science<br>Bakolis, Ioannis; King's College London<br>Healey, Andy; King's College London Institute of Psychiatry Psychology and Neuroscience, Centre for Implementation Science; King's College London, King's Health Economics<br>Harrington, Jean; King's College London, Department of Psychological Medicine<br>Woods, Anthony; King's College London, Department of Psychological Medicine<br>Crane, Nikki; King's College London<br>Jones, Fiona; St Georges University of London<br>Pariante, Carmine; King's College London, Psychological Medicine<br>Sevdalis, Nick; King's College London

Keywords:	Stroke < NEUROLOGY, QUALITATIVE RESEARCH, STATISTICS & RESEARCH METHODS

SCHOLARONE™
Manuscripts

Evaluation of a community-based performance arts programme for people that have experienced stroke – protocol for the SHAPER-Stroke Odysseys study

Authors

Joint first authors: Carolina Estevao and Maria Baldellou Lopez

Carolina Estevao[1], Maria Baldellou Lopez[2], Rachel Davis[2], Lucinda Jarrett[3], Tayana Soukup[2], Ioannis Bakolis[2,4], Andy Healy[2,6], Jean Harrington[1], Anthony Woods[1], Nikki Crane[7], Fiona Jones[8], Carmine Pariante[1], Nick Sevdalis[2]

[1]Department of Psychological Medicine, Institute of Psychiatry, Psychology and Neuroscience, King's College London, 5 Cutcombe Rd, Brixton, London SE5 9RT, United Kingdom
[2]Centre for Implementation Science, Health Service and Population Research Department, Institute of Psychiatry, Psychology & Neuroscience, 16 De Crespigny Park, Camberwell, London SE5 8AB, United Kingdom
[3]Rosetta Life Head Office, 3 Brook End, Chadlington, Chipping Norton, OX7 3NF, United Kingdom
[4]Department of Biostatistics and Health Informatics, Institute of Psychiatry, Psychology & Neuroscience, 16 De Crespigny Park, Camberwell, London SE5 8AB, United Kingdom
[5]King's Health Economics, Health Service and Population Research Department, Institute of Psychiatry, Psychology & Neuroscience, 16 De Crespigny Park, Camberwell, London SE5 8AB, United Kingdom
[6]King's Health Economics, Health Service and Population Research Department, Institute of Psychiatry, Psychology & Neuroscience, 16 De Crespigny Park, Camberwell, London SE5 8AB, United Kingdom
[7]Culture team, King's College London, Somerset House East Wing, Strand WC2R 2LS, United Kingdom
[8] Faculty of Health, Social Care and Education, Kingston University and St George's, University of London, London, United Kingdom

Corresponding author: Carolina Estevao[1], carolina.estevao@kcl.ac.uk

Keywords: Performance, stroke, art, community-based, implementation science, dance, poetry, song, music

Word count: 3,584 words

Abstract

Introduction: Stroke survivors once in the community face challenges with their long-term rehabilitation care and present higher levels of loneliness, depression and anxiety than the rest of the population. A community-based performance arts programme, Stroke Odysseys (SO), has been devised to tackle the challenges of stroke

For peer review only

survivors. In this study we aim to evaluate the implementation, impact and experiences of (SO for stroke survivors).

Methods: This study, SHAPER-SO, within the Scaling-up Health Arts Programmes: Implementation and Effectiveness Research (SHAPER) programme, aims to scale-up Stroke Odysseys to 75 participants and 47 ambassadors, while simultaneously evaluating the effectiveness and implementation of the study. The study will also recruit 47 wider stakeholders involved in the referral, delivery or facilitation of SO.

Ethics and dissemination: Ethical approval has been granted by the King's College London PNM Research Ethics Panel, REC reference: LRS/DP-20/21-21549. Clinical Trials.gov: NCT04864470

For peer review only

Article Summary

Strengths and limitations

- First study examining an arts intervention on stroke survivors, using a two-pronged evaluation
- The unique study design will result in a package of clinical and implementation effectiveness data on this particular intervention.
- There may be inconsistency in participant experience throughout an intervention period if in-person sessions are switched online and vice-versa due to COVID-19 social distancing restrictions.

Introduction

Stroke affects over 113,000 people every year [1] and, according to the latest statistics, there are currently more than 1.2 million stroke survivors in the UK [2,3]. The effects of stroke are often devastating, with almost two-thirds of survivors leaving hospital with a disability and half experiencing depression within five years [4,5]. In addition to the substantial impact stroke has on those affected and their caregivers, it can also pose a significant financial burden to health and social care services. The societal cost of stroke has been estimated to be £26 billion per annum, with NHS costs accounting for £3.4 billion in 2015, and projected to increase to £10.2 billion by 2035 [6].

Stroke survivors commonly face emotional, social and psychological challenges, with depression, anxiety and apathy being the most prevalent neuropsychiatric sequelae [7]. Such disabling symptoms are often coupled by feelings of abandonment [8] once hospital rehabilitation ends and their recovery plateaus. Stroke survivors in the UK usually receive rehabilitation whilst in hospital but once they are discharged, the level of support in the community tends to be variable and in the long term, inadequate for their needs [8]. This puts significant pressure on caregivers which are then relied upon as the main support system throughout recovery. It is therefore no surprise that caregivers commonly experience anxiety and depression [9], with prevalence rates ranging between 30%-45% and 20–50% [10], respectively. This is consistent with a meta-review of qualitative systematic reviews [11] which reported a lack of self-management resources available following stroke, highlighting the gap between available services and the long-term social, emotional and physical needs of stroke survivors throughout their rehabilitation journey [12]. Consistent with this are the findings of a survey by the Stroke Association in the UK, which emphasised the devastating burden and 'hidden effects' of stroke [13] . The survey, which collated data from over 10,000 stroke survivors, is the biggest to date in the UK and revealed the effects of stroke on cognition, emotions, relationships and mental health are widespread, can be life-long, and are often overlooked

or neglected. In the survey, 50% of stroke survivors and 85% of caregivers reported a gap between the support provided versus the support they felt was needed. While current stroke rehabilitation targets functional recovery, it fails to meet psychosocial needs of stroke survivors.

The evidence summarised above suggests that there is a need for more holistic rehabilitation programmes, especially non-pharmacological and non-invasive modalities, to address the psychosocial needs and improve quality of life of stroke survivors [14]. Arts-based programmes (such as 'Stroke Odysseys' discussed below) are one such approach that show promising results in enhancing the wellbeing, self-esteem, social life and rehabilitation experiences of stroke patients [15].

Stroke Odysseys

Rosetta Life, a well-established non-profit organisation with a track record of conducting arts programmes for stroke and brain injury survivors in hospital, developed Stroke Odysseys (SO), a performance- based arts programme, with continued consultation from stakeholders, including stroke survivors. SO provides an opportunity for those who have had a stroke or brain injury to share their experiences to an audience through movement, music, song-writing and the spoken word. The programme, which has now been running for over 21 years, uses performance arts to help stroke survivors overcome psychological challenges such as lowered self-esteem, anxiety and depression, which are commonly reported by individuals [16].

In this protocol paper, we present our plans to evaluate and scale-up SO. This study is embedded in the Scaling-up Health-Arts Programme: Implementation and Effectiveness Research (SHAPER) research programme, which is, to our knowledge, the world's largest programme on arts and health that aims to translate community-based arts programmes into to implementable and scalable health interventions in the healthcare system [17]. SHAPER-SO will be a two-pronged study, examining the implementation and clinical effectiveness of this intervention. The research we will be undertaking examines both the impact of the arts on mental health and also how SO can be embedded into clinical pathways. To the best of our knowledge, SHAPER-SO is the first study of its kind in the context of stroke care and rehabilitation.

Aims and objectives

The three main objectives in this study are: (1) to explore the clinical impact of SO on stroke survivors; (2) to explore SO implementation aspects including uptake, adoption, perceived acceptability, appropriateness, feasibility, fidelity of receipt, unintended consequences, and sustainability; and (3) to evaluate implementation costs and cost-effectiveness of the intervention, with focus on the costs associated with implementing SO into existing care pathways, health services, partner organisations and commissioning and the impact of scaling up SO on the utilisation of health services.

The main research aim is to evaluate the implementation, impact and experiences of a community-based performance arts programme (SO for stroke survivors).

Our study objectives are as follows:

1. To explore impact of participation in performance programmes for people that have experienced stroke.
2. To study the context, mechanisms of delivery and interactions between participants and facilitators which take place during SO delivery.

For peer review only

3. To explore learning and experiences of facilitators and participants after
4. To evaluate any change in the emotional wellbeing, participation and activity of stroke participants pre and post-SO
5. To evaluate the extent to which SO is acceptable, feasible to undertake and appropriate to survivors and wider stakeholders (including ambassadors, artists and clinician referrers to the programme).
6. To assess any unintended consequences of SO.
7. To explore the challenges, barriers and facilitators to the implementation of SO.
8. To assess the costs associated with the implementation of the programme.
9. To explore the strategies used within individual sites to implement the programme.
10. To assess the adoption, adherence to it and attrition rates of the programme.

Theoretical underpinning

An ethnographic and constructivist approach will be used to examine stroke survivors' experiences of the SO programme. This is described as the study of social interactions, behaviours and perceptions that occurs within groups, team organisation and communities. Ethnography provides rich, holistic insights into people's views and actions, as well as the nature of the location (context) they inhabit. The aim has been described as 'getting inside' the way each group of people sees the world [17]. Ethnography has a strong emphasis on 'unstructured data and involves implicit interpretation of the meaning and function of human interactions, rather than hypothesis testing. This approach aligns well with the complex nature of the Stroke Odysseys programme.

The implementation analyses are informed by several well-established implementation science frameworks, which we have applied to develop a set of implementation facets of SO to assess, both quantitatively and qualitatively (see Methods). We used the recently developed 'Implementation Science Research Development' (ImpRes) framework [18] to identify the elements of implementation that the study ought to capture, ImpRes defined 10 different domain that an implementation evaluation ought to capture – including capturing stakeholder engagement, outcome of implementation (e.g., how acceptable, appropriate and feasible SO and its implementation processes are to those delivering and also receiving SO) and any unintended consequences. Moreover, we reviewed the COM-B tool [19] to help us identify any barriers that may affect individual's engagement with the SO programme; the Consolidated Framework for Implementation Research (CFIR) [20,21] to help us map reported barriers and drivers to the implementation of the SO; and finally the Reach Effectiveness Adoption Implementation Maintenance (RE-AIM) model [22,23] taken together with Proctor et al's [24] taxonomy of implementation outcomes, guided our choice of implementation measures to assess.

Methods and analysis

Design

SHAPER-SO is a mixed-methods programme study, comprising quantitative and qualitative methods to assess the clinical and implementation outcomes outlined in the measures section below.

The intervention

Stroke Odysseys comprises three distinct stages 1) weekly workshops conducted over 12 weeks for stroke participants which will be facilitated by an integrated team of expert artists and 'stroke ambassadors' from the

For peer review only

charity Rosetta Life, 2) a smaller group of ambassadors recruited from the workshops will be trained to become co-facilitators (i.e. new stroke ambassadors), 3) a performance tour including education and taster workshops for audiences.

During sessions, which run for three hours each, participants devise a dance and music performance work from their own stories. The practice of "performing ourselves" is key to achieving successful outcomes such as transforming the participants' perception of identity. The culmination of the programme will be a public facing performance to an audience of carers, health care practitioners, friends, family and the wider community.

Due to the ongoing COVID19 pandemic and the necessity of shielding of vulnerable adults and foreseeing increased anxiety in stroke survivors to attend in-person sessions, we have adapted the SO programme to be delivered through a mixture of live/face-to-face and online delivery (blended approach). Participants will be able to choose whether to attend the sessions/participate face-to-face or online based on their personal preferences and needs. The researcher will manage groups to ensure that all the participants that wish to attend in person will be able to do so during the 12-weeks.

The adapted programme will still be run in three stages, described below:

The live groups will alternate so that as many participants as possible will be able to experience the three parts of the process – (1) building the company, (2) gathering material and devising, and (3) rehearsing and performance.

Participants will be able to choose whether to attend the sessions/participate face-to-face or online based on their personal preferences and needs.

Stage 2) After the performance is completed, participants will be invited to a four-day training programme where they will learn to act as advocates for life after stroke – termed 'stroke ambassadors'. The programme will take place once weekly and will be led by a team of artists and supported by a leadership coach. All training will take place on Zoom until social distancing measures are lifted, and participants are willing to meet indoors – a blended ambassador training will be offered.

Stage 3) Following training, a volunteer manager will coordinate a tailored programme where ambassadors support artists in recruitment, befriend the newly discharged stroke-survivors and take part in small scale performance tours to challenge the perception of disability. The tour will be delivered online with online screenings followed by Q&A with ambassadors, taster sessions and exercises delivered online with the ambassadors.

The programme will be delivered in two cycles of the complete 3-stage intervention. At the end of the two cycles of programme, a group of newly trained ambassadors will emerge. The programme seeks to develop a national network of Ambassadors who will build capacity for performance arts in healthcare and wider capacity for healthcare. The Stroke Ambassadors are graduates of the 12-week workshop that receive training, based on a leadership-coaching model, and they deliver a tailored advocacy programme according to their creative skills – befriending, performance administration and support, programme advocacy.

Study Setting

The study will take place online until conditions of the pandemic enable researchers, artists and participants to meet safely indoors, as per government guidelines. When it is feasible and safe to meet in person, participants,

For peer review only

artists and researchers will meet in an established performance arts education centre in order to ensure that COVID guidelines on cleanliness are guaranteed.

Sample and Recruitment

Stroke survivors

Consenting stroke participants will be included if they are:

(1) over 18 years of age
(2) have had one or more stroke(s)
(3) received inpatient care in a UK stroke care pathway
(4) able to follow a 2-stage command and hold a conversation in English if no supporter/friend is available to translate

The following exclusion criteria will be applied to individuals:

(1) with co-morbidities that would prevent participation in group activities e.g. dementia or deteriorating or fluctuating palliative conditions
(2) unable to understand English
(3) unable to commit to the 12-week programme

Stroke ambassadors will be included if they have been through the ambassador training and involved in at least one programme cycle culminating in the tour.

Wider stakeholder group

In addition to the stroke survivors that enrol on the SO programme, data will also be collected from a wider stakeholder group involved in the delivery or support of the programme. Individuals will be recruited if they meet the following criteria:

(1) over 18 years of age
(2) can hold a conversation in English if no supporter/friend is available to translate
(3) can either be defined as a:
    - Supporters: family members or carers.
    - Deliverers: individuals responsible for the delivery of the research (facilitators and artists).
    - Referrers: individuals involved in signposting (e.g. doctors, nurses, healthcare workers).

Wider stakeholders will be excluded from participation if they are unable to understand English or if no supporter/friend is available to translate.

Sampling

Size of sample

We aim to recruit 75 new stroke survivors in total for the duration of the study. Based on previous experience of running SO where participants then complete an ambassador training cycle, a drop-out rate of 20% is expected and so the final number of ambassadors that complete the ambassador training is estimated to be 60.

For peer review only

The wider stakeholder group will be recruited from the network of people who are involved in the programme and present in the community. This includes the voluntary sector, health and social care sectors and clinical commissioners. A total of 47 stakeholders, a forecast based on the existing network numbers, will be recruited (12 carers, 10 clinical team members, 5 artists, 20 existing ambassadors).

Recruitment procedure

Potential stroke survivor participants will be identified through signposting in community centres and care homes as well as engaging in presentations, screenings, taster sessions and performances during the tour.

Recruitment will be done online. Screenings of performance extracts will be followed by taster sessions online and a Q&A with ambassadors. Potential participants will be directed to the project manager at Rosetta Life who will manage all referrals.

Wider stakeholders will be recruited from the networks of people involved in the referral, delivery or supporting of the programme.

Study Flowchart

A study overview can be seen in the flowchart below (Figure 1):

Data collection

This is a prospective mixed methods study using a range of qualitative and quantitative methods at different time points pre-, during and post- intervention of each programme cycle.

Qualitative methods will comprise semi-structured interviews and non-participant observations of training and production to assess experiences and attitudes towards the programme and the implementation of it.

Quantitative methods will be used to assess experiences and attitudes towards the SO programme and its implementation. A clinical measure OX-PAQ will be used to assess participation and activity in stroke survivors. Further information on these measures is included further in the 'methods' section in the outcome measures tables (Tables 1 and 2) and 'assessment descriptions' section.

Outcomes

Data on the clinical outcomes will be collected from stroke survivors who have enrolled on the SO programme (see Table 1). Data on the implementation outcomes will be collected from stroke survivors who have enrolled on the SO programme as well as the wider stakeholder group involved in the SO programme, including deliverers, referrers, and supporters (see Table 2).

For peer review only

Table 1. Clinical outcomes

Objective	Clinical Outcome Measures/Endpoints	Type of assessment	Time point for data collection
**Primary objective**			
To evaluate emotional wellbeing, participation and activity of stroke participants and any change pre and post SO programme	Ox-PAQ (Oxford Participation and Activities Questionnaire, 23-item, patient-reported outcome measure)	Quantitative	T0 and T2
**Secondary objectives**			
To study the context, mechanisms and interactions which take place during Stroke Odysseys delivery	Non-participant observations of workshops	Qualitative	T1 (during workshop delivery)
To explore learning and experiences of facilitators and participants	Semi structured interviews- stroke participants and facilitators	Qualitative	T2
To explore stroke survivors' preparation and participation in performances	Semi structured interviews- stroke participants	Qualitative	T0

Note: data on the clinical outcomes will be collected from stroke survivors who have enrolled on the SO programme

Objective	Implementation Outcome Measures/Endpoints	Type of assessment	Time points for data collection	Who data will be collected from
Primary objective				
To evaluate to what extent Stroke Odysseys is acceptable, to survivors and wider stakeholders	Acceptability of intervention Measure (AIM)<br><br>Semi-structured interviews (to explore reasons for acceptability score)	Quantitative<br><br>Qualitative	T1, T2, T3<br><br>T2, T3	Stroke survivors, deliverers, supporters, referrers

For peer review only - http://bmjopen.bmj.com/site/about/guidelines.xhtml

				Stroke survivors, deliverers, supporters, referrers
Secondary objectives				
To evaluate to what extent Stroke Odysseys is appropriate to survivors and wider stakeholders	Intervention Appropriateness Measure (IAM)<br><br>Semi-structured interviews (to explore reasons for appropriateness score)	Quantitative<br><br>Qualitative	T1, T2, T3<br><br>T2, T3	Stroke survivors, deliverers, supporters, referrers<br><br>Stroke survivors, deliverers, supporters, referrers
To evaluate to what extent Stroke Odysseys feasible to survivors and wider stakeholders	Feasibility Intervention Measure (FIM)<br>Semi-structured interviews (to explore reasons for feasibility score)	Quantitative<br><br>Qualitative	T1, T2, T3<br><br>T2, T3	Stroke survivors, deliverers, supporters, referrers<br><br>Stroke survivors, deliverers, supporters, referrers
To assess any unintended consequences of the programme	Semi-structured interviews	Qualitative	T2, T3	Stroke survivors, deliverers, supporters, referrers
To explore the facilitators and barriers to implementing the programme	Semi-structured interviews	Qualitative	T2, T3	Stroke survivors, deliverers, supporters, referrers
To explore the facilitators and barriers to sustained use of the programme	Semi-structured interviews	Qualitative	T2, T3	Stroke survivors, deliverers, supporters, referrers
To assess service utilisation and cost associated costs and changes in quality of life associated with the implementation of the programme	EQ5D-5L (quality of life measure) and AD-SUS (adult service receipt schedule) and semi structured interviews and activity data (to estimate implementation costs).	Quantitative	T2 and T3	Stroke survivors<br><br>Stroke survivors, deliverers, supporters, referrers

To explore the strategies including resource inputs utilised, used within individual sites to implement the programme	Semi-structured interviews	Qualitative	T2, T3	Deliverers, referrers
To assess the adoption of the programme	The number of individuals delivering the programme, and the number of individuals supporting the programme (and continuing to do so)	Quantitative	T0, T2, T3	Deliverers, referrers
To assess programme adherence and attrition rates	Data on the overall adherence to the programme, number of drops-outs and reasons why	Quantitative<br><br>Qualitative	Data recorded from register on weekly attendance rates for 12-week programme (stage 1) and for 4-week ambassador programme (stage 2)<br><br>T2, T3	Deliverers (record data)<br><br>Stroke survivors

Table 2. Implementation outcomes

Notes: Data on the implementation outcomes will be collected from stroke survivors who have enrolled on the SO programme as well as the wider stakeholder group involved in the SO programme (including deliverers, referrers and supports).

Timepoints for data collection: T0 – Baseline; T1- Midway through the 12-week programme (weeks 5-7); T2 - Immediately post performance (12-14 weeks); T3 - Immediately after the advocacy training for stroke ambassadors.

For peer review only

To maximise inclusivity and outcome completion, and minimise participant burden, outcome assessments, where possible, will be conducted either face-to-face, online, by telephone or via postal questionnaire depending on the outcome measures being assessed, participants preferences and government COVID-19 guidelines.

Assessment descriptions for clinical outcomes

Qualitative assessments

Ethnographic research

Ethnographic non-participant observations of a selection of the 12 workshops including at least 1-2 groups from each of the two phases (building confidence, rehearsal and production) to capture facilitator and participant practice, interactions and routines. Each observation period will last for the duration of the workshop, and the researcher will record field-notes contemporaneously.

Semi-structured interviews

Semi-structured interviews will be held with facilitators and participants pre- and post-programme cycles to explore anticipated concerns and expectations (pre) and experiences of facilitation and factors influencing delivery, engagement of participants, adaptation and learning (post).

Quantitative assessment (Ox-PAQ)

The Oxford Participation and Activities Questionnaire (Ox-PAQ) is a 23-item, fully FDA compliant [25] patient reported outcome measure developed specifically to assess participation and activity in individuals with chronic health problems including those with neurological conditions such as Subarachnoid Haemorrhage, Motor Neurone Disease, Multiple Sclerosis and Parkinson's disease. Ox-PAQ items have been generated using the World Health Organisation (WHO) International Classification of Functioning, Disability and Health (ICF) theoretical framework. Participation is reflected across three domains, namely Routine Activities (14 items), Emotional Well-Being (5 items) and Social Engagement (4 items), all of which demonstrate sound psychometric properties in terms of validity, reliability and sensitivity to change with effect sizes ranging from 0.28 (social engagement) to 0.44 (emotional well-being) [25].

Assessment descriptions for the implementation outcomes

Quantitative assessments

Validated and standardised implementation scales will be used to gather quantitative data on how acceptable, appropriate and feasible the SO programme is perceived by stroke survivors, ambassadors, deliverers, supporters and referrers. These scales include the Acceptability of Programme Measure (AIM), the Programme Appropriateness Measure (IAM) and the Feasibility of Programme Measure (FIM. For further information on the development of these scales, please refer to the paper by Weiner et al [26].

The investigators will quantify and cost the resources used in implementing the programme, evaluate wider service utilisation and associated costs before and after participants complete the programme, including any changes to their quality-of-life profile measured using the EQ5D-3L preference-based QoL measure.

For peer review only

Qualitative data collection

Semi-structured interviews

Semi-structured interviews will be conducted with a purposive sub-sample of stroke survivors to explore their attitudes towards the acceptability, appropriateness and feasibility of the programme, as well as factors (facilitators or barriers) that affected their involvement (and potential drop-out) and any unintended consequences. These issues will also be explored with a sub-sample of individuals from each of the wider stakeholder groups (10 in total).

Interview guides have been based on existing implementation frameworks (see above) and adapted from a previous project [27]. They will be further adapted and co-designed with our stakeholder group to ensure the questions in the interview guide are meaningful and address the core aims of the study.

Interviews will be audio taped and are anticipated to be conducted 1:1 or in participants dyads, face to face (government guidelines permitting) or remotely by phone or video.

Data Analysis

Data will be analysed using quantitative and qualitative approaches.

Quantitative analysis

Descriptive statistics of survey data will be performed (frequency distribution, central tendency). Parametric and non-parametric tests will also be employed to compare the survey responses to the OX-PAQ, AIM, FIM, IAM and EQ5D before and after the SO intervention. Changes in OX-PAQ, AIM, FIM, IAM and EQ5D will be assessed using generalised linear models depending on the distribution of the outcome (continuous, binary, ordinal). All analyses will be conducted in STATA V.14.1.

Qualitative analysis

Initial analysis of qualitative data will be undertaken using an inductive thematic approach. All data from interviews and observations will be managed using NVivo 10 and examined to categorise themes and key issues that emerge. Using this inductive approach, tentative theoretical explanations will be generated for each sub-group. Summary memos for data sets will be developed for each sub-group to provide the basis for within and between group comparisons.

CFIR (www.CFIR.org) will be used to further guide the coding and analysis (i.e. framework analysis) of interview data to identify barriers and facilitators to the implementation and sustainment of SO programme. This approach has been used previously, that is, CFIR has been applied post-implementation to investigate facilitators and barriers to implementation among stakeholders who had already adopted and implemented an innovation, thus identifying determinants of implementation post-hoc [28,29].

Reflective summaries: The relationship of the researcher(s) with the research context they are investigating will be presented in the form of a written narrative of ideas and experiences during data collection. These reflective summaries will be shared with the research team and externally to judge any possible biases with the way the data was collected or prior assumptions.

For peer review only

Patient & public involvement

The programme has been developed and further refined using co design methodologies with a group of 20 members of South London stroke communities. The project has been shared widely with stroke clinicians across London and has their full support. During the pandemic Rosetta Life set up an advisory group consisting of Stroke Ambassadors to support the redesign of the website www.strokeodysseys.org, to monitor how people living with the effects of a stroke were engaging with the online workshops, to oversee the development of the education videos and the Ambassadors Handbook.

This advisory group is now a stable and national network of Ambassadors who curate an online programme and advise on the development and delivery of SO. They have advised the investigators on the need to ensure that the measures were aphasia friendly and found an organisation to make sure that the measures were aphasia friendly. They will now look at the language of the Implementation Science measures and make sure that they are accessible.

Trial Registration details

This study is registered on ClinicalTrials.gov PRS under the ClinicalTrials.gov ID: NCT04864470.

Ethics and dissemination

Ethical approval has been granted by the King's College London PNM Research Ethics Panel, REC reference: LRS/DP-20/21-21549

Informed consent will be collected from all research participants and stakeholders involved in the study.

Findings will be published in peer-reviewed journals and disseminated at national and international meetings.

References

1 Rothwell PM, Coull AJ, Giles MF, *et al.* Change in stroke incidence, mortality, case-fatality, severity, and risk factors in Oxfordshire, UK from 1981 to 2004 (Oxford Vascular Study). *Lancet* 2004;**363**:1925–33. doi:10.1016/S0140-6736(04)16405-2

2 National Institute for Health and Care Excellence. NICE impact stroke. 2019. https://www.nice.org.uk/Media/Default/About/what-we-do/Into-practice/measuring-uptake/NICE-Impact-stroke.pdf (accessed 23 Jul 2021).

For peer review only

3 Stroke Association. Stroke statistics. Stroke Association. 2020.https://www.stroke.org.uk/what-is-stroke/stroke-statistics (accessed 23 Jul 2021).

4 Adamson J, Beswick A, Ebrahim S. Is stroke the most common cause of disability? *J Stroke Cerebrovasc Dis* 2004;**13**:171–7. doi:10.1016/j.jstrokecerebrovasdis.2004.06.003

5 Ayerbe L, Ayis S, Crichton S, *et al.* The long-term outcomes of depression up to 10 years after stroke; the South London Stroke Register. *J Neurol Neurosurg Psychiatry* 2014;**85**:514–21. doi:10.1136/jnnp-2013-306448

6 King D, Wittenberg R, Patel A, *et al.* The future incidence, prevalence and costs of stroke in the UK. *Age and Ageing* 2020;**49**:277–82. doi:10.1093/ageing/afz163

7 Ferro JM, Caeiro L, Figueira ML. Neuropsychiatric sequelae of stroke. *Nat Rev Neurol* 2016;**12**:269–80. doi:10.1038/nrneurol.2016.46

8 Pindus DM, Mullis R, Lim L, *et al.* Stroke survivors' and informal caregivers' experiences of primary care and community healthcare services - A systematic review and meta-ethnography. *PLoS One* 2018;**13**:e0192533. doi:10.1371/journal.pone.0192533

9 Berg A, Palomäki H, Lönnqvist J, *et al.* Depression Among Caregivers of Stroke Survivors. *Stroke* 2005;**36**:639–43. doi:10.1161/01.STR.0000155690.04697.c0

10 Zhao J, Zeng Z, Yu J, *et al.* Effect of main family caregiver's anxiety and depression on mortality of patients with moderate-severe stroke. *Sci Rep* 2021;**11**:2747. doi:10.1038/s41598-021-81596-8

11 Pearce G, Pinnock H, Epiphaniou E, *et al.* Experiences of Self-Management Support Following a Stroke: A Meta-Review of Qualitative Systematic Reviews. *PLOS ONE* 2015;**10**:e0141803. doi:10.1371/journal.pone.0141803

12 Roads to Recovery - Organisation and quality of stroke services. NIHR Evidence. doi:10.3310/themedreview-001685

13 Lived Experience of Stroke. Stroke Association 2019. http://www.stroke.org.uk/sites/default/files/conferences/nisc/documents/lived_experience_of_stroke_chapter_1.pdf

14 Lo TLT, Lee JLC, Ho RTH. Creative Arts-Based Therapies for Stroke Survivors: A Qualitative Systematic Review. *Front Psychol* 2018;**9**:1646. doi:10.3389/fpsyg.2018.01646

15 Chan CKP, Lo TLT, Wan AHY, *et al.* A randomised controlled trial of expressive arts-based intervention for young stroke survivors. *BMC Complementary Medicine and Therapies* 2021;**21**:7. doi:10.1186/s12906-020-03161-6

16 Barker-Collo SL. Depression and anxiety 3 months post stroke: prevalence and correlates. *Arch Clin Neuropsychol* 2007;**22**:519–31. doi:10.1016/j.acn.2007.03.002

17 Estevao C, Fancourt D, Dazzan P, *et al.* Scaling-up Health-Arts Programmes: the largest study in the world bringing arts-based mental health interventions into a national health service. *BJPsych Bulletin* 2021;**45**:32–9. doi:10.1192/bjb.2020.122

For peer review only

18 Hull L, Goulding L, Khadjesari Z, *et al.* Designing high-quality implementation research: development, application, feasibility and preliminary evaluation of the implementation science research development (ImpRes) tool and guide. *Implementation Science* 2019;**14**:80. doi:10.1186/s13012-019-0897-z

19 Michie S, van Stralen MM, West R. The behaviour change wheel: A new method for characterising and designing behaviour change interventions. *Implementation Science* 2011;**6**:42. doi:10.1186/1748-5908-6-42

20 The Consolidated Framework for Implementation Research. https://cfirguide.org/tools/ (accessed 21 Sep 2021).

21 Damschroder LJ, Aron DC, Keith RE, *et al.* Fostering implementation of health services research findings into practice: a consolidated framework for advancing implementation science. *Implementation Science* 2009;**4**:50. doi:10.1186/1748-5908-4-50

22 RE-AIM – Reach Effectiveness Adoption Implementation Maintenance. https://www.re-aim.org/ (accessed 21 Sep 2021).

23 Glasgow RE, Vogt TM, Boles SM. Evaluating the public health impact of health promotion interventions: the RE-AIM framework. *Am J Public Health* 1999;**89**:1322–7. doi:10.2105/ajph.89.9.1322

24 Proctor E, Silmere H, Raghavan R, *et al.* Outcomes for Implementation Research: Conceptual Distinctions, Measurement Challenges, and Research Agenda. *Adm Policy Ment Health* 2011;**38**:65–76. doi:10.1007/s10488-010-0319-7

25 Morley D, Dummett S, Kelly L, *et al.* Measuring improvement in health-status with the Oxford Participation and Activities Questionnaire (Ox-PAQ). *PROM* 2019;**Volume 10**:153–6. doi:10.2147/PROM.S198619

26 Weiner BJ, Lewis CC, Stanick C, *et al.* Psychometric assessment of three newly developed implementation outcome measures. *Implementation Science* 2017;**12**:108. doi:10.1186/s13012-017-0635-3

27 Soukup T, Hull L, Smith EL, *et al.* Effectiveness-implementation hybrid type 2 trial evaluating two psychoeducational programmes for severe hypoglycaemia in type 1 diabetes: implementation study protocol. *BMJ Open* 2019;**9**:e030370. doi:10.1136/bmjopen-2019-030370

28 Kirk MA, Kelley C, Yankey N, *et al.* A systematic review of the use of the Consolidated Framework for Implementation Research. *Implementation Science* 2016;**11**:72. doi:10.1186/s13012-016-0437-z

29 Proctor E, Silmere H, Raghavan R, *et al.* Outcomes for Implementation Research: Conceptual Distinctions, Measurement Challenges, and Research Agenda. *Adm Policy Ment Health* 2011;**38**:65–76. doi:10.1007/s10488-010-0319-7

Figure legends:

Figure 1 SHAPER-SO study flowchart.

For peer review only - http://bmjopen.bmj.com/site/about/guidelines.xhtml

Author statement

All authors listed have contributed to the conception and design of the protocol and this manuscript. All authors have been involved in the drafting of the manuscript and have individually approved the version of the work published.

Specifically, the contribution of each author falls within the following CRediT categories:

CE, MBL, RD, TS, IB, JH, AH, FJ and NS: conceptualization, methodology and project administration.

AW, NC, CP: conceptualisation, project administration and funding acquisition.

LJ: conceptualisation, project administration.

Funding

This trial is part of the SHAPER programme, a Scaling-up Health-Arts Programme to scale up arts programmes. This programme is funded by the Wellcome Trust (award reference 219425/Z/19/Z). This work is additionally supported by the National Institute for Health Research (NIHR) Biomedical Research Centre at South London and Maudsley NHS Foundation Trust and King's College London and by a NIHR Senior Investigator to CMP.

Conflicts of Interest

NS is the director of the London Safety and Training Solutions Ltd, which offers training in patient safety, implementation solutions and human factors to healthcare organisations and the pharmaceutical industry.

CMP reports grants from Wellcome Trust, during the conduct of the study; grants from National Institute for Health Research (NIHR), grants from NIHR Senior Investigator, grants from Johnson & Johnson, grants from Wellcome Trust, outside the submitted work.

The other authors have no conflicts of interest to declare.

Data availability

Non applicable, data has not been generated yet. Recruitment to start in the Autumn 2021.

For peer review only

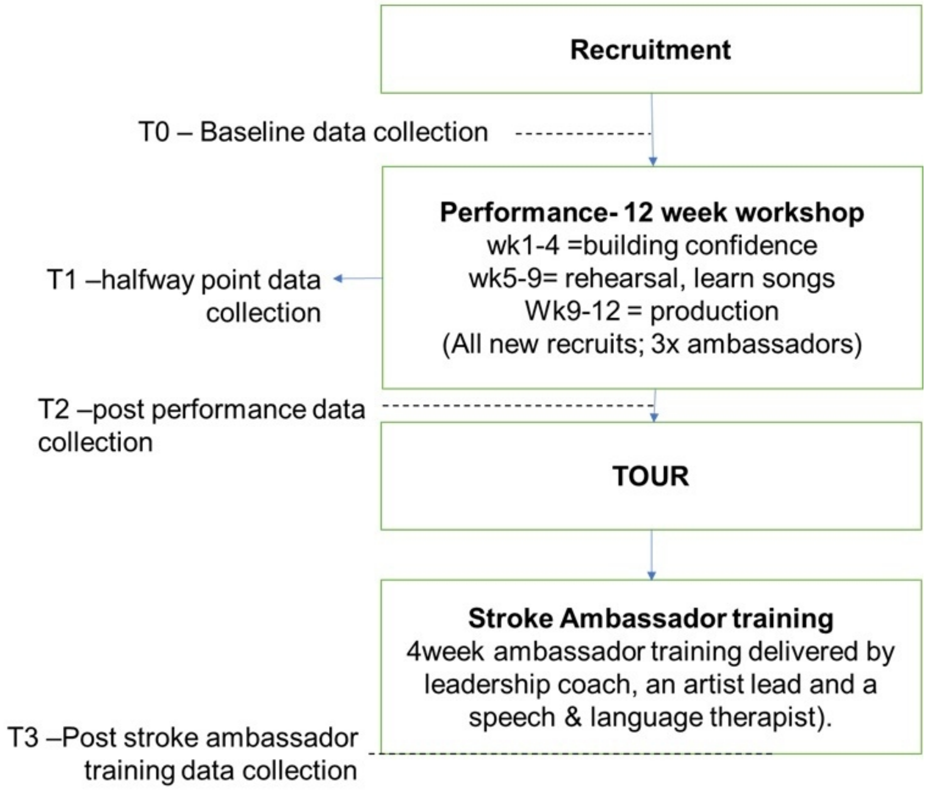

SHAPER-SO flowchart

144x119mm (144 x 144 DPI)

BMJ Open

Evaluation of a community-based performance arts programme for people that have experienced stroke in the UK – protocol for the SHAPER-Stroke Odysseys study

Journal:	*BMJ Open*
Manuscript ID	bmjopen-2021-057805.R1
Article Type:	Protocol
Date Submitted by the Author:	25-Jan-2022
Complete List of Authors:	Estevao, Carolina; King's College London, nstitute of Psychiatry, Psychology & Neuroscience<br>Baldellou Lopez, Maria ; King's College London Institute of Psychiatry Psychology and Neuroscience, Centre for Implementation Science<br>Davis, Rachel; Florence Nightingale School of Nursing, James Clerk Maxell Building<br>Jarret, Lucinda; Rosetta Life Head Office<br>Soukup, Tayana ; King's College London, Centre Implementation Science<br>Bakolis, Ioannis; King's College London<br>Healey, Andy; King's College London Institute of Psychiatry Psychology and Neuroscience, Centre for Implementation Science; King's College London, King's Health Economics<br>Harrington, Jean; King's College London, Department of Psychological Medicine<br>Woods, Anthony; King's College London, Department of Psychological Medicine<br>Crane, Nikki; King's College London<br>Jones, Fiona; St Georges University of London<br>Pariante, Carmine; King's College London, Psychological Medicine<br>Fancourt, Daisy; University College London Research Department of Epidemiology and Public Health, Department of Behavioural Science and Health<br>Sevdalis, Nick; King's College London
**Primary Subject Heading**:	Qualitative research
Secondary Subject Heading:	Rehabilitation medicine, Research methods, Occupational and environmental medicine, Neurology
Keywords:	Stroke < NEUROLOGY, QUALITATIVE RESEARCH, STATISTICS & RESEARCH METHODS

SCHOLARONE™
Manuscripts

Evaluation of a community-based performance arts programme for people that have experienced stroke in the UK – protocol for the SHAPER-Stroke Odysseys study

Authors

Joint first authors: Carolina Estevao and Maria Baldellou Lopez

Carolina Estevao[1], Maria Baldellou Lopez[2], Rachel Davis[2], Lucinda Jarrett[3], Tayana Soukup[2], Ioannis Bakolis[2,4], Andy Healy[2,5], Jean Harrington[1], Anthony Woods[1], Nikki Crane[6], Fiona Jones[7], Carmine Pariante[1], Daisy Fancourt[8], Nick Sevdalis[2]

[1]Department of Psychological Medicine, Institute of Psychiatry, Psychology and Neuroscience, King's College London, 5 Cutcombe Rd, Brixton, London SE5 9RT, United Kingdom
[2]Centre for Implementation Science, Health Service and Population Research Department, Institute of Psychiatry, Psychology & Neuroscience, 16 De Crespigny Park, Camberwell, London SE5 8AB, United Kingdom
[3]Rosetta Life Head Office, 3 Brook End, Chadlington, Chipping Norton, OX7 3NF, United Kingdom
[4]Department of Biostatistics and Health Informatics, Institute of Psychiatry, Psychology & Neuroscience, 16 De Crespigny Park, Camberwell, London SE5 8AB, United Kingdom
[5]King's Health Economics, Health Service and Population Research Department, Institute of Psychiatry, Psychology & Neuroscience, 16 De Crespigny Park, Camberwell, London SE5 8AB, United Kingdom
[6]Culture team, King's College London, Somerset House East Wing, Strand WC2R 2LS, United Kingdom
[7]Faculty of Health, Social Care and Education, Kingston University and St George's, University of London, London, United Kingdom
[8]Department of Behavioural Science and Health, University College London, Gower Street, London, WC1E 6BT, United Kingdom

Corresponding author: Carolina Estevao[1], carolina.estevao@kcl.ac.uk

Keywords: Performance, stroke, art, community-based, implementation science, dance, poetry, song, music

Word count: 5,120 words

Abstract

Introduction: Stroke survivors, once in the community, face challenges with their long-term rehabilitation care and present higher levels of loneliness, depression, and anxiety than the rest of the population. A community-based performance arts programme, Stroke Odysseys (SO), has been devised to tackle the challenges of living with stroke in the UK. In this study, we aim to evaluate the implementation, impact, and experiences of SO for stroke survivors.

Methods: SHAPER-SO aims to scale-up Stroke Odysseys to 75 participants and 47 stakeholders, while simultaneously evaluating the effectiveness and implementation of the study. The study will evaluate the experience and impact of Stroke Odysseys on those participating using mixed methods (interviews, observations and surveys) before and after each stage, and carry out non-participant observations during a percentage of the

For peer review only

workshops, training and tour. This is a study within the Scaling-up Health Arts Programmes: Implementation and Effectiveness Research (SHAPER) programme.

Ethics and dissemination: Ethical approval has been granted by the King's College London PNM Research Ethics Panel, REC reference: LRS/DP-20/21-21549. Clinical Trials.gov: NCT04864470.

Strengths and limitations

- The first study examining an art intervention on stroke survivors, using a two-pronged evaluation.
- The unique study design will result in a package of clinical and implementation data on this particular intervention.
- There may be inconsistency in participant experience throughout an intervention period if in-person sessions are switched online and vice-versa due to COVID-19 social distancing restrictions.

1. Introduction

Stroke affects over 113,000 people every year [1] and, according to the latest statistics, there are currently more than 1.2 million stroke survivors in the UK [2,3]. The effects of stroke are often devastating, with almost two-thirds of survivors leaving the hospital with a disability and half experiencing depression within five years [4,5]. In addition to the substantial impact stroke has on those affected and their caregivers, it can also pose a significant financial burden to health and social care services. The societal cost of stroke has been estimated to be £26 billion per annum, with NHS costs accounting for £3.4 billion in 2015, and projected to increase to £10.2 billion by 2035 [6].

Stroke survivors commonly face emotional, social and psychological challenges, with depression, anxiety and apathy being the most prevalent neuropsychiatric sequelae [7]. Such disabling symptoms are often coupled with feelings of abandonment [8] once hospital rehabilitation ends and their recovery plateaus. Stroke survivors in the UK usually receive rehabilitation whilst in hospital but once they are discharged, the level of support in the community tends to be variable and in the long-term, inadequate for their needs [8]. This is consistent with a meta-review of qualitative systematic reviews [9] which reported a lack of self-management resources available following stroke, highlighting the gap between available services and the long-term social, emotional and physical needs of stroke survivors throughout their rehabilitation journey [10]. Additionally, the findings of a survey by the Stroke Association in the UK, emphasised the devastating burden and 'hidden effects' of stroke [11]. The survey, which collated data from over 10,000 stroke survivors and is the biggest to date in the UK, revealed that the effects of stroke on cognition, emotions, relationships, and mental health are widespread, can be life-long, and are often overlooked or neglected. In the survey, 50% of stroke survivors and 85% of caregivers reported a gap between the support provided versus the support they felt was needed. While current stroke rehabilitation targets functional recovery, it fails to meet the psychosocial needs of stroke survivors.

The evidence summarised above suggests that there is a need for more holistic rehabilitation programmes, especially non-pharmacological and non-invasive modalities, to address the psychosocial needs and improve the quality of life of stroke survivors [12]. Arts-based programmes (such as 'Stroke Odysseys' discussed below) are one such approach that shows promising results in enhancing the wellbeing, self-esteem, social life and rehabilitation experiences of stroke patients [13]. Indeed, over the past decade, several studies conducted in this patient population have consistently shown a positive impact of different art modalities on psychological (e.g.

For peer review only

enhancement in confidence and a better sense of control), social (e.g. increased social interactions and peer support) and functional (e.g. improvement in physical abilities) outcomes [12].

Nonetheless, despite the growing body of research on the benefits of art interventions, the process of scaling-up these interventions, embedding them into healthcare and its associated challenges are not yet well-established. Preliminary data indicates that Stroke Odysseys (discussed below) is received positively by those who take part [14], however, identifying barriers to implementation and exploring ways to overcome these obstacles is essential to successfully and sustainably embed SO into clinical pathways and roll out the programme at a wider scale.

Stroke Odysseys is part of the Scaling-up Health-Arts Programme: Implementation and Effectiveness Research (SHAPER), which is, to our knowledge, the world's largest study on arts and health examining both clinical effectiveness and implementation effectiveness of three community-based arts programmes: Melodies for Mums (M4M), a singing intervention for postnatal depression, PD-Ballet, a dance intervention for Parkinson's Disease, and Stroke Odysseys (SO). Overall, SHAPER has three primary aims: (1) to successfully embed each of the art interventions into the healthcare system (i.e. taking a social prescribing approach), (2) to scale up these interventions at a larger scale, and (3) to facilitate these interventions being commissioned by CCGs, ensuring the long-term sustainability of delivery [15].

1.1. Stroke Odysseys

Rosetta Life, a well-established non-profit organisation with a track record of conducting arts programmes for stroke and brain injury survivors, developed Stroke Odysseys (SO), a performance-based arts programme with continued consultation from stakeholders (including stroke survivors). SO provides an opportunity for those who have had a stroke or brain injury to share their experiences with an audience through movement, music, songwriting and the spoken word. The programme, which has now been running for over 21 years, uses performance arts to help stroke survivors overcome psychological challenges such as lowered self-esteem, anxiety and depression, which are commonly reported by individuals [16].

In this protocol paper, we present our plans to evaluate. SHAPER-SO will be a two-pronged study, examining the implementation and clinical effectiveness of Stroke Odysseys. The research we will be undertaking examines both, the impact of performance arts on participants and how SO can be embedded into clinical pathways. This will help us to identify not just 'if' but also 'why' the programme works and support our understanding of how it can be successfully delivered and scaled up within clinical pathways. Alongside this, we will examine participants' experiences of the programme using an ethnographic and constructivist approach. To the best of our knowledge, SHAPER-SO is the first study of its kind in the context of stroke care and rehabilitation.

2. Aims and objectives

The three main objectives in this study are: (1) to explore the clinical impact (effectiveness) of SO on stroke survivors; (2) to explore SO implementation aspects including uptake, adoption, perceived acceptability, appropriateness, feasibility, the fidelity of receipt, unintended consequences, and sustainability; and (3) to evaluate implementation costs and cost-effectiveness of the intervention, with focus on the costs associated with implementing SO into existing care pathways, health services, partner organisations and commissioning and the impact of scaling up SO on the utilisation of health services.

For peer review only

The main research aim is to evaluate the implementation, effectiveness, impact and experiences of a community-based performance arts programme (SO for stroke survivors).

Our study objectives are as follows:

1. To explore the impact of participation in performance programmes on cognitive health and physical, psychological and social well-being of people that have experienced stroke.
2. To study the context, mechanisms of delivery and interactions between participants and facilitators which take place during SO delivery.
3. To explore the learning and experiences of facilitators and participants after SO delivery.
4. To evaluate any change in the emotional wellbeing, participation, and activity of stroke participants pre- and post-SO
5. To evaluate the extent to which SO is acceptable, feasible to undertake and appropriate to survivors and wider stakeholders (including ambassadors, artists, and clinician referrers to the programme).
6. To, explore the challenges, barriers, facilitators and unintended consequences of the implementation of SO.
7. To assess the costs associated with the implementation of the programme.
8. To, assess the adoption, adherence to it and attrition rates of the programme.

3. Theoretical underpinning

An ethnographic and constructivist approach will be used to examine stroke survivors' experiences of the SO programme (objective 2). This is described as the study of social interactions, behaviours and perceptions that occurs within groups, team organisation and communities. Ethnography provides rich, holistic insights into people's views and actions, as well as the nature of the location (context) they inhabit. The aim has been described as 'getting inside' the way each group of people sees the world [17]. Ethnography has a strong emphasis on 'unstructured data and involves implicit interpretation of the meaning and function of human interactions, rather than hypothesis testing. This approach aligns well with the complex nature of the Stroke Odysseys programme.

The implementation analyses are informed by several well-established implementation science frameworks, which we have applied to develop a set of implementation facets of SO to assess, both quantitatively and qualitatively (see Methods). We used the recently developed 'Implementation Science Research Development' (ImpRes) framework [17] to identify the elements of implementation that the study ought to capture, ImpRes defined 10 different domains that an implementation evaluation ought to capture – including capturing stakeholder engagement, the outcome of implementation (e.g., how acceptable, appropriate and feasible SO and its implementation processes are to those delivering and also receiving SO) and any unintended consequences (objective 3, 5 and 6). Moreover, we reviewed the Capability, Opportunity, and Motivation Model of Behaviour (COM-B) tool [18] to help us identify any barriers that may affect an individual's engagement with the SO programme (objectives 7 and 10). The COM-B components lie at the centre of the Behaviour Change Wheel (BCW), a framework for designing and characterising behaviour change interventions [18]. The Consolidated Framework for Implementation Research (CFIR) [19,20] will help us map reported barriers and drivers to the implementation of the SO (objective 7); and finally, the Reach Effectiveness Adoption Implementation Maintenance (RE-AIM) model [21,22] taken together with Proctor et al's [23] taxonomy of implementation outcomes, guided our choice of implementation measures to assess.

4. Methods and analysis

4.1. Design

For peer review only - http://bmjopen.bmj.com/site/about/guidelines.xhtml

SHAPER-SO is a mixed-methods programme study, comprising quantitative and qualitative methods to assess the clinical and implementation outcomes outlined in the measures section below.

4.2. The intervention

Stroke Odysseys is a post-stroke performance art intervention designed and delivered by arts organisation Rosetta Life. This intervention initially developed and funded by King's and Guy's & St Thomas' Charity, aims to improve recovery, agency and well-being after stroke [14].

Stroke Odysseys comprises three distinct stages 1) weekly workshops conducted over 12 weeks for stroke participants which will be facilitated by an integrated team of expert artists and 'stroke ambassadors' from the charity Rosetta Life, 2) a smaller group of ambassadors recruited from the workshops will be trained to become co-facilitators (i.e. new stroke ambassadors), 3) a performance tour including education and taster workshops for audiences.

During sessions, which run for three hours each, participants devise a dance and music performance work from their own stories. The practice of "performing ourselves" is key to achieving successful outcomes such as transforming the participants' perception of identity. The culmination of the programme will be a public-facing performance to an audience of carers, health care practitioners, friends, family and the wider community.

Due to the ongoing COVID-19 pandemic and the necessity of shielding vulnerable adults and foreseeing increased anxiety in stroke survivors to attend in-person sessions, we have adapted the SO programme to be delivered through a mixture of live/face-to-face and online delivery (blended approach). Participants will be able to choose whether to attend the sessions/participate face-to-face or online based on their personal preferences and needs. The researcher will manage groups to ensure that all the participants that wish to attend in person will be able to do so during the 12-weeks.

The adapted programme will still be run in three stages, described below:

Stage 1) The workshops are the result of co-creation; the general framework is: weeks 1 - 3 building the performance company, weeks 4 - 6 devising the performance weeks 6 - 9 rehearsing the performance and weeks 10 - 12 are sometimes concertina-ed into one production week introducing stage management, lighting and technical runs. Each of the 12 workshops contains a performance "class" of 20 - 30mins exploring movement and voice techniques and exercise.

Participants will be able to choose whether to attend the sessions/participate face-to-face or online based on their personal preferences and needs.

Stage 2) After the performance is completed, participants will be invited to a four-day training programme where they will learn to act as advocates for life after stroke – termed 'stroke ambassadors'. The optional ambassador training starts with an introduction to being an ambassador and an outline of the pathways available: a) supporting artists in hospital and community contexts, b) speaking the press and media / advocating for life after stroke, c) engaging in academic research and d) joining the steering group that informs activities and directions. The skills development training is delivered in three stages: an introduction to movement practices and the traditions of independent dance, then an introduction to voice and improvisation and finally, an introduction to performance. Each ambassador then constructs an individually tailored programme according to their personal goals and intentions in becoming an ambassador.

The programme will take place once weekly and will be led by a team of artists and supported by a leadership coach. All training will take place on Zoom until social distancing measures are lifted, and participants are willing to meet indoors – a blended ambassador training will be offered.

Stage 3) Following training, a volunteer manager will coordinate a tailored programme where ambassadors support artists in recruitment, befriend the newly discharged stroke survivors and take part in small scale performance tours to challenge the perception of disability. The tour will be delivered online with online screenings followed by Q&A with ambassadors, taster sessions and exercises delivered online with the ambassadors.

For peer review only - http://bmjopen.bmj.com/site/about/guidelines.xhtml

The programme will be delivered in two cycles of the complete 3-stage intervention. At the end of the two cycles of the programme, a group of newly trained ambassadors will emerge. The programme seeks to develop a national network of Ambassadors who will build capacity for performance arts in healthcare and a wider capacity for healthcare. The Stroke Ambassadors are graduates of the 12-week workshop that receive training, based on a leadership-coaching model, and they deliver a tailored advocacy programme according to their creative skills – befriending, performance administration and support, programme advocacy.

4.3. Study Setting

The study will take place online until conditions of the pandemic enable researchers, artists, and participants to meet safely indoors, as per government guidelines. When it is feasible and safe to meet in person, participants, artists, and researchers will meet in an established performance arts education centre to ensure that COVID guidelines on cleanliness are guaranteed. When ran in person, the workshops are run in Central London locations, with a single centre running the programme in each cycle.

4.4. Sample and Recruitment

Stroke survivors

Consenting stroke participants will be included if they are:

(1) over 18 years of age
(2) have had one or more stroke(s)
(3) received inpatient care in a UK stroke care pathway
(4) able to follow a 2-stage command and hold a conversation in English if no supporter/friend is available to translate

The following exclusion criteria will be applied to individuals:

(1) with co-morbidities that would prevent participation in group activities (e.g. dementia or deteriorating or fluctuating palliative conditions)
(2) unable to understand English
(3) unable to commit to the 12-week programme

Additionally, stroke ambassadors will be included if they have been through the ambassador training and are involved in at least one programme cycle culminating in the tour.

All participants will be offered the option of completing an interview with their carer present. This will be offered both after the first 12-week programme and after the ambassador training, for those that wish to participate. Those that decline will be asked if they would be willing to provide their reasons why.

Wider stakeholder group

In addition to the stroke survivors that enrol on the SO programme, data will also be collected from a wider stakeholder group involved in the delivery or support of the programme. Individuals will be recruited if they meet the following criteria:

(1) over 18 years of age
(2) can hold a conversation in English if no supporter/friend is available to translate
(3) can either be defined as a:
    - Supporters: family members or carers.
    - Deliverers: individuals responsible for the delivery of the research (facilitators and artists).
    - Referrers: individuals involved in signposting (e.g. doctors, nurses, healthcare workers).

For peer review only

Wider stakeholders will be excluded from participation if they are unable to understand English or if no supporter/friend is available to translate.

4.5. Sampling

4.5.1. Size of sample

We aim to recruit 75 new stroke survivors in total for the duration of the study. A prediction of 75 participants has been estimated based on the numbers that over the years running Stroke Odysseys, *Rosetta Life* has been able to recruit in two consecutive cycles. This number has also considered the organisation being able to whilst maintain a manageable ratio of participants to artists and staff members, guaranteeing that Stroke Odysseys is delivered to the highest standard.

Based on previous experience of running SO where participants then complete an ambassador training cycle, a drop-out rate of 20% is expected and so the final number of ambassadors that complete the ambassador training is estimated to be 60.

The wider stakeholder group will be recruited from the network of people who are involved in the programme and present in the community. This includes the voluntary sector, health and social care sectors and clinical commissioners. A total of 47 stakeholders, a forecast based on the existing network numbers, will be recruited (12 carers, 10 clinical team members, 5 artists, 20 existing ambassadors).

4.5.2. Recruitment procedure

Potential stroke survivor participants will be identified through signposting in community centres and care homes as well as engaging in presentations, screenings, taster sessions and performances during the tour.

Recruitment of potential participants will be done online. Screenings of performance extracts will be followed by taster sessions online and a Q&A with ambassadors. Potential participants will be directed to the project manager at Rosetta Life who will manage all referrals.

Potential participants will be offered a PIS and an ICF and will be explained the details of the study. Written consent will be sought following a 48h colling-off period.

Wider stakeholders will be recruited from the networks of people involved in the referral, delivery or support of the programme.

A recruitment log will be kept by the research team to accurately record included and excluded participants as well as missing data from dropouts to account for possible sampling bias.

5. Study Flowchart

A study overview can be seen in the flowchart below (Figure 1):

6. Data collection

This is a prospective mixed-methods study using a range of qualitative and quantitative methods at different time points pre-, during and post-intervention of each programme cycle.

Qualitative methods will comprise semi-structured interviews and non-participant observations of training and production to assess experiences and attitudes towards the programme and its implementation.

Quantitative methods will be used to assess experiences and attitudes towards the SO programme and its implementation. Further information is included further in the 'methods' section in the outcome measures tables (Tables 1 and 2) and the 'assessment descriptions' section.
Demographic data will be collected by Rosetta Life at the time of enrolment.

6.1. Outcomes

Data on the clinical outcomes will be collected from stroke survivors who have enrolled on the SO programme (see Table 1). Data on the implementation outcomes will be collected from stroke survivors who have enrolled on the SO programme as well as the wider stakeholder group involved in the SO programme, including deliverers, referrers, and supporters (see Table 2).

For peer review only

Table 1. Clinical outcomes

Objective	Clinical Outcome Measures/Endpoints	Type of assessment	The time point for data collection
**Primary objective**			
**Secondary objectives**			
To study the context, mechanisms and interactions which take place during Stroke Odysseys delivery	Non-participant observations of workshops	Qualitative	T1 (during workshop delivery)
To explore the learning and experiences of facilitators and participants	Semi-structured interviews- stroke participants and facilitators	Qualitative	T2
To explore stroke survivors' preparation and participation in performances	Semi-structured interviews- stroke participants	Qualitative	T0

Note: Data on the clinical outcomes will be collected from stroke survivors who have enrolled on the SO programme

Table 2. Implementation outcomes

Objective	Implementation Outcome Measures/Endpoints	Type of assessment	Time points for data collection	Who data will be collected from
Primary objective				
To evaluate to what extent Stroke Odysseys is acceptable, to survivors and wider stakeholders	Acceptability of intervention Measure (AIM)<br><br>Semi-structured interviews (to explore reasons for acceptability score)	Quantitative<br><br>Qualitative	T1, T2, T3<br><br>T2, T3	Stroke survivors, deliverers, supporters, referrers<br><br>Stroke survivors, deliverers, supporters, referrers
Secondary objectives				
To evaluate to what extent Stroke Odysseys are appropriate to survivors and wider stakeholders	Intervention Appropriateness Measure (IAM)<br><br>Semi-structured interviews (to explore reasons for appropriateness score)	Quantitative<br><br>Qualitative	T1, T2, T3<br><br>T2, T3	Stroke survivors, deliverers, supporters, referrers<br><br>Stroke survivors, deliverers, supporters, referrers

For peer review only

To evaluate to what extent Stroke Odysseys feasible to survivors and wider stakeholders	Feasibility Intervention Measure (FIM)<br>Semi-structured interviews (to explore reasons for feasibility score)	Quantitative<br><br>Qualitative	T1, T2, T3<br><br>T2, T3	Stroke survivors, deliverers, supporters, referrers<br><br>Stroke survivors, deliverers, supporters, referrers
To assess any unintended consequences of the programme	Semi-structured interviews	Qualitative	T2, T3	Stroke survivors, deliverers, supporters, referrers
To explore the facilitators and barriers to implementing the programme	Semi-structured interviews	Qualitative	T2, T3	Stroke survivors, deliverers, supporters, referrers
To explore the facilitators and barriers to sustained use of the programme	Semi-structured interviews	Qualitative	T2, T3	Stroke survivors, deliverers, supporters, referrers
To assess service utilisation and cost associated costs and changes in quality of life associated with the implementation of the programme	EQ5D-5L (quality of life measure) and AD-SUS (adult service receipt schedule) and semi-structured interviews and activity data (to estimate implementation costs).	Quantitative	T2 and T3	Stroke survivors<br><br>Stroke survivors, deliverers, supporters, referrers
To explore the strategies including resource inputs utilised, used within individual sites to implement the programme	Semi-structured interviews	Qualitative	T2, T3	Deliverers, referrers
To assess the adoption of the programme	The number of individuals delivering the programme, and the number of individuals supporting the programme (and continuing to do so)	Quantitative	T0, T2, T3	Deliverers, referrers
To assess programme adherence and attrition rates	Data on the overall adherence to the programme, number of drops-outs and reasons why	Quantitative<br><br>Qualitative	Data recorded from the register on weekly attendance rates for the 12-week programme (stage 1) and 4-week ambassador programme (stage 2)<br><br>T2, T3	Deliverers (record data)<br><br>Stroke survivors

Notes: Data on the implementation outcomes will be collected from stroke survivors who have enrolled on the SO programme as well as the wider stakeholder group involved in the SO programme (including deliverers, referrers and supports).

For peer review only

Timepoints for data collection: T0 - Baseline; T1 - Midway through the 12-week programme (weeks 5-7); T2 - Immediately post-performance (12-14 weeks); T3 - Immediately after the advocacy training for stroke ambassadors.

For peer review only

To maximise inclusivity and outcome completion, and minimise participant burden, outcome assessments, where possible, will be conducted either face-to-face, online, by telephone or via postal questionnaire depending on the outcome measures being assessed, participants preferences and government COVID-19 guidelines.

6.2. Assessment descriptions for clinical outcomes

Qualitative assessments

6.2.1. Ethnographic research

Ethnographic non-participant observations of a selection of the 12 workshops including at least 1-2 groups from each of the two phases (building confidence, rehearsal and production) to capture facilitator and participant practice, interactions and routines. Each observation period will last for the duration of the workshop, and the ethnographic researcher will record field notes contemporaneously.

6.2.2. Semi-structured interviews

Semi-structured interviews will be held with facilitators and participants pre- and post-programme cycles to explore anticipated concerns and expectations (pre) and experiences of facilitation and factors influencing delivery, engagement of participants, adaptation, and learning (post).
The implementation science research team will be interviewing participants across both, the 12-week programme and ambassador training, in addition to wider stakeholders.

6.3. Assessment descriptions for the implementation outcomes

6.3.1. Quantitative assessments

Validated and standardised implementation scales will be used to gather quantitative data on how acceptable, appropriate, and feasible the SO programme is perceived by stroke survivors, ambassadors, deliverers, supporters and referrers. These scales include the Acceptability of Programme Measure (AIM), the Programme Appropriateness Measure (IAM) and the Feasibility of Programme Measure (FIM. For further information on the development of these scales, please refer to the paper by Weiner et al [24].
The implementation science researchers will quantify and cost the resources used in implementing the programme, evaluate wider service utilisation and associated costs before and after participants complete the programme, including any changes to their quality-of-life profile measured using the EQ5D-3L preference-based QoL measure. The EQ5D-3L is a self-complete multi-attribute measure of health-related quality of life that assigns individuals a unique state of health based on their response to individual items. Each unique health state is associated with a pre-determined "utility" value derived from a survey of wider community preferences over different states of health. The utility-scale is anchored at 1 (full health) and zero (death), with negative values allowed in instances where states of health are considered worse than death. Health state utility values are subsequently used to estimate quality-adjusted years survived over time (QALYs) – the utility scores providing the means of making the quality adjustments. Evidence on costs and QALYs will subsequently be used to inform an analysis of the cost-effectiveness of programme delivery at scale.

6.3.2. Qualitative data collection

Semi-structured interviews

For peer review only

Semi-structured interviews will be conducted with a purposive sub-sample of stroke survivors (N= 20: 5 from each cycle at two-time points – T2 and T3). Interviews will be carried out with this sub-sample of stroke survivors to explore their attitudes towards the acceptability, appropriateness, and feasibility of the programme, as well as factors (facilitators or barriers) that affected their involvement (and potential drop-out) and any unintended consequences. These issues will also be explored with a sub-sample of individuals (10 in total) from each of the wider stakeholder groups.

Interview guides have been based on existing implementation frameworks (see above) and adapted from a previous project [25]. They will be further adapted and co-designed with our stakeholder group to ensure the questions in the interview guide are meaningful and address the core aims of the study.

Interviews will be audiotaped and are anticipated to be conducted 1:1 or in participants dyads, face to face (government guidelines permitting) or remotely by phone or video.

7. Data Analysis

Data will be analysed using quantitative and qualitative approaches.

7.1. Quantitative analysis

Descriptive statistics of survey data will be performed (frequency distribution, central tendency). Parametric and non-parametric tests will also be employed to compare the survey responses to the AIM, FIM, IAM and EQ5D before and after the SO intervention. Changes in AIM, FIM, IAM and EQ5D will be assessed using generalised linear models depending on the distribution of the outcome (continuous, binary, ordinal). All analyses will be conducted in STATA V.14.1.

7.2. Qualitative analysis

Initial analysis of qualitative data will be undertaken using an inductive approach to thematic analysis. All data from interviews and observations will be managed using NVivo 10 and examined to categorise themes and key issues that emerge. Using this inductive approach, tentative theoretical explanations will be generated for each sub-group. Summary memos for data sets will be developed for each sub-group to provide the basis for within and between-group comparisons. The inductive approach is data-driven; based on observation, the early analysis seeks to reveal patterns and themes from which tentative hypothesis can be drawn subsequently leading to theory; theories are devised to explain what is seen rather than the other way around.

CFIR (www.CFIR.org) will be used to further guide the coding and analysis (i.e. framework analysis) of interview data to identify barriers and facilitators to the implementation and sustainment of the SO programme. This approach has been used previously, that is, CFIR has been applied post-implementation to investigate facilitators and barriers to implementation among stakeholders who had already adopted and implemented an innovation, thus identifying determinants of implementation posthoc [26,27].

Reflective summaries: The relationship of the researcher(s) with the research context they are investigating will be presented in the form of a written narrative of ideas and experiences during data collection. These reflective summaries will be shared with the research team and externally to judge any possible biases with the way the data was collected or prior assumptions.

For peer review only

8. Patient & Public involvement

The programme has been developed and further refined using co-design methodologies with a group of 20 members of South London stroke communities. The project has been shared widely with stroke clinicians across London and has their full support. During the pandemic Rosetta Life set up an advisory group consisting of Stroke Ambassadors to support the redesign of the website www.strokeodysseys.org, to monitor how people living with the effects of a stroke were engaging with the online workshops, to oversee the development of the education videos and the Ambassadors Handbook.

This advisory group is now a stable and national network of Ambassadors who curate an online programme and advise on the development and delivery of SO. They have advised the investigators on the need to ensure that the measures were aphasia friendly and found an organisation to make sure that the measures were aphasia friendly. They will now look at the language of the Implementation Science measures and make sure that they are accessible.

9. Trial Registration details

This study is registered on ClinicalTrials.gov PRS under the ClinicalTrials.gov ID: NCT04864470.

10. Ethics and dissemination

Ethical approval has been granted by the King's College London PNM Research Ethics Panel, REC reference: LRS/DP-20/21-21549. Informed consent will be collected from all research participants and stakeholders involved in the study. Findings will be published in peer-reviewed journals and disseminated at national and international meetings.

11. Data Protection

The Investigator will ensure that this study is conducted in full conformity with relevant regulations and with the ICH Guidelines for Good Clinical Practice (CPMP/ICH/135/95) July 1996. The Investigator will ensure that this study is conducted in accordance with the principles of the Declaration of Helsinki.

Access to person identifiable implementation science data will rest with the data custodian(s) from the immediate study team and the implementation science team. Since the project seeks to explore in some depth participants' experiences and barriers and facilitators to implementation, it is important to maintain strict confidentiality and facilitate openness in the interviews and survey responses thus optimal data quality.

Consent forms and audio/video recordings will be kept electronically in KCL's SharePoint for the duration of the study, only accessible by the teams at KCL, Kingston University and Rosetta Life involved in the study. Consent forms and other identifiable paperwork will be kept in locked cabinets only accessible to the study team. Study data will be kept in a separate location from the person identifiable information. Access to the de-identified research data will be shared with the study management group for the purposes of review, analysis and dissemination. Only de-identified data will be analysed.

After the completion of the study, the study data will be kept for the King's College London's standard retention period of 10 years after the completion of the study. The study data that supports published

For peer review only

results will be deposited in a secure data repository (e.g. King's Research Data Management System). This will allow the data to be accessible for future reuse as per King's College London's policy on the management of research data long-term.

12.References

1 Rothwell PM, Coull AJ, Giles MF, *et al.* Change in stroke incidence, mortality, case-fatality, severity, and risk factors in Oxfordshire, UK from 1981 to 2004 (Oxford Vascular Study). *Lancet* 2004;**363**:1925–33. doi:10.1016/S0140-6736(04)16405-2

2 National Institute for Health and Care Excellence. NICE impact stroke. 2019. https://www.nice.org.uk/Media/Default/About/what-we-do/Into-practice/measuring-uptake/NICE-Impact-stroke.pdf (accessed 23 Jul 2021).

3 Stroke Association. Stroke statistics. Stroke Association. 2020.https://www.stroke.org.uk/what-is-stroke/stroke-statistics (accessed 23 Jul 2021).

4 Adamson J, Beswick A, Ebrahim S. Is stroke the most common cause of disability? *J Stroke Cerebrovasc Dis* 2004;**13**:171–7. doi:10.1016/j.jstrokecerebrovasdis.2004.06.003

5 Ayerbe L, Ayis S, Crichton S, *et al.* The long-term outcomes of depression up to 10 years after stroke; the South London Stroke Register. *J Neurol Neurosurg Psychiatry* 2014;**85**:514–21. doi:10.1136/jnnp-2013-306448

6 King D, Wittenberg R, Patel A, *et al.* The future incidence, prevalence and costs of stroke in the UK. *Age and Ageing* 2020;**49**:277–82. doi:10.1093/ageing/afz163

7 Ferro JM, Caeiro L, Figueira ML. Neuropsychiatric sequelae of stroke. *Nat Rev Neurol* 2016;**12**:269–80. doi:10.1038/nrneurol.2016.46

8 Pindus DM, Mullis R, Lim L, *et al.* Stroke survivors' and informal caregivers' experiences of primary care and community healthcare services - A systematic review and meta-ethnography. *PLoS One* 2018;**13**:e0192533. doi:10.1371/journal.pone.0192533

9 Pearce G, Pinnock H, Epiphaniou E, *et al.* Experiences of Self-Management Support Following a Stroke: A Meta-Review of Qualitative Systematic Reviews. *PLOS ONE* 2015;**10**:e0141803. doi:10.1371/journal.pone.0141803

10 Roads to Recovery - Organisation and quality of stroke services. NIHR Evidence. doi:10.3310/themedreview-001685

11 Lived Experience of Stroke. Stroke Association 2019. http://www.stroke.org.uk/sites/default/files/conferences/nisc/documents/lived_experience_of_stroke_chapter_1.pdf

12 Lo TLT, Lee JLC, Ho RTH. Creative Arts-Based Therapies for Stroke Survivors: A Qualitative Systematic Review. *Front Psychol* 2018;**9**:1646. doi:10.3389/fpsyg.2018.01646

13 Chan CKP, Lo TLT, Wan AHY, *et al.* A randomised controlled trial of expressive arts-based intervention for young stroke survivors. *BMC Complementary Medicine and Therapies* 2021;**21**:7. doi:10.1186/s12906-020-03161-6

For peer review only

14 Jarrett L. Stroke Odysseys: performance arts after stroke, a service innovation for neurological rehabilitation. *British Journal of Neuroscience Nursing* 2018;**14**:S18–21. doi:10.12968/bjnn.2018.14.Sup5.S18

15 Estevao C, Fancourt D, Dazzan P, *et al.* Scaling-up Health-Arts Programmes: the largest study in the world bringing arts-based mental health interventions into a national health service. *BJPsych Bulletin* 2021;**45**:32–9. doi:10.1192/bjb.2020.122

16 Barker-Collo SL. Depression and anxiety 3 months post-stroke: prevalence and correlates. *Arch Clin Neuropsychol* 2007;**22**:519–31. doi:10.1016/j.acn.2007.03.002

17 Hull L, Goulding L, Khadjesari Z, *et al.* Designing high-quality implementation research: development, application, feasibility and preliminary evaluation of the implementation science research development (ImpRes) tool and guide. *Implementation Science* 2019;**14**:80. doi:10.1186/s13012-019-0897-z

18 Michie S, van Stralen MM, West R. The behaviour change wheel: A new method for characterising and designing behaviour change interventions. *Implementation Science* 2011;**6**:42. doi:10.1186/1748-5908-6-42

19 The Consolidated Framework for Implementation Research. https://cfirguide.org/tools/ (accessed 21 Sep 2021).

20 Damschroder LJ, Aron DC, Keith RE, *et al.* Fostering implementation of health services research findings into practice: a consolidated framework for advancing implementation science. *Implementation Science* 2009;**4**:50. doi:10.1186/1748-5908-4-50

21 RE-AIM – Reach Effectiveness Adoption Implementation Maintenance. https://www.re-aim.org/ (accessed 21 Sep 2021).

22 Glasgow RE, Vogt TM, Boles SM. Evaluating the public health impact of health promotion interventions: the RE-AIM framework. *Am J Public Health* 1999;**89**:1322–7. doi:10.2105/ajph.89.9.1322

23 Proctor E, Silmere H, Raghavan R, *et al.* Outcomes for Implementation Research: Conceptual Distinctions, Measurement Challenges, and Research Agenda. *Adm Policy Ment Health* 2011;**38**:65–76. doi:10.1007/s10488-010-0319-7

24 Weiner BJ, Lewis CC, Stanick C, *et al.* Psychometric assessment of three newly developed implementation outcome measures. *Implementation Science* 2017;**12**:108. doi:10.1186/s13012-017-0635-3

25 Soukup T, Hull L, Smith EL, *et al.* Effectiveness-implementation hybrid type 2 trial evaluating two psychoeducational programmes for severe hypoglycaemia in type 1 diabetes: implementation study protocol. *BMJ Open* 2019;**9**:e030370. doi:10.1136/bmjopen-2019-030370

26 Kirk MA, Kelley C, Yankey N, *et al.* A systematic review of the use of the Consolidated Framework for Implementation Research. *Implementation Science* 2016;**11**:72. doi:10.1186/s13012-016-0437-z

27 Proctor E, Silmere H, Raghavan R, *et al.* Outcomes for Implementation Research: Conceptual Distinctions, Measurement Challenges, and Research Agenda. *Adm Policy Ment Health* 2011;**38**:65–76. doi:10.1007/s10488-010-0319-7

For peer review only

Figure titles:

Figure 1: SHAPER-SO study flowchart

13.Contributors

All authors listed have contributed to the conception and design of the protocol and this manuscript. All authors have been involved in the drafting of the manuscript and have individually approved the version of the work published. Specifically, the contribution of each author falls within the following CRediT categories: CE, MBL, RD, TS, IB, JH, AH, FJ, DF and NS: conceptualization, methodology and project administration. AW, NC, CP: conceptualisation, project administration and funding acquisition. LJ: conceptualisation, project administration.

14.Funding

This trial is part of the SHAPER programme, a Scaling-up Health-Arts Programme to scale up arts programmes. This programme is funded by the Wellcome Trust (award reference 219425/Z/19/Z). This work is additionally supported by the National Institute for Health Research (NIHR) Biomedical Research Centre at South London and Maudsley NHS Foundation Trust and King's College London and by an NIHR Senior Investigator to CMP.

15.Conflicts of Interest

NS is the director of the London Safety and Training Solutions Ltd, which offers training in patient safety, implementation solutions and human factors to healthcare organisations and the pharmaceutical industry. CMP reports grants from Wellcome Trust, during the conduct of the study; grants from National Institute for Health Research (NIHR), grants from NIHR Senior Investigator, grants from Johnson & Johnson, grants from Wellcome Trust, outside the submitted work. The other authors have no conflicts of interest to declare.

For peer review only

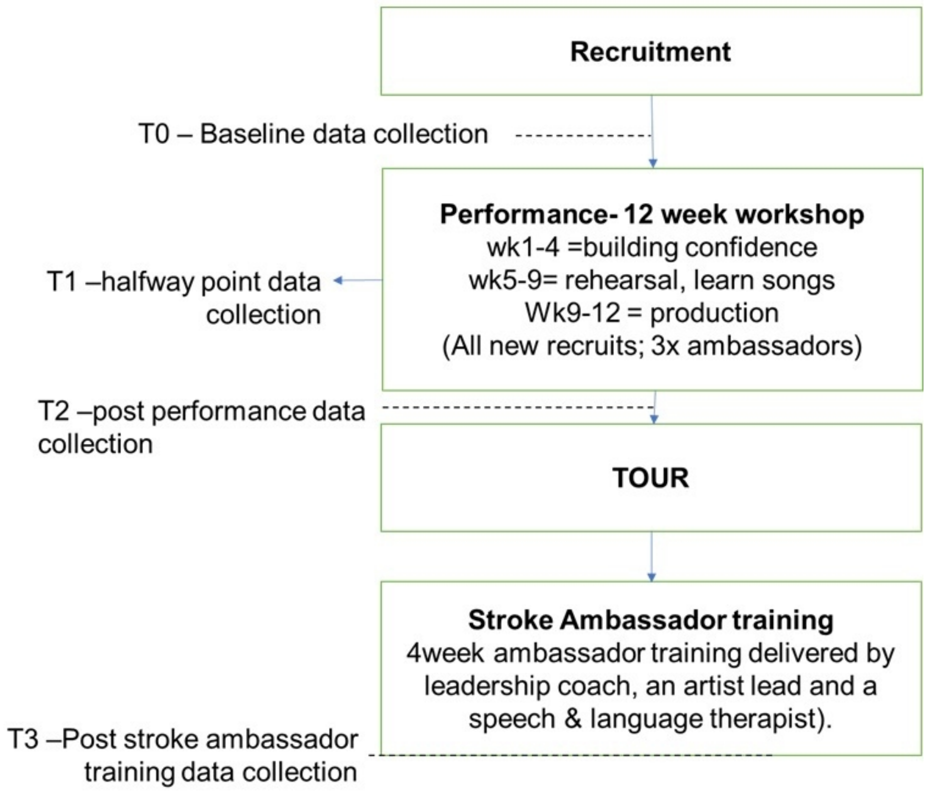

SHAPER-SO flowchart

144x119mm (144 x 144 DPI)

BMJ Open

Evaluation of a community-based performance arts programme for people who have experienced stroke in the UK: protocol for the SHAPER-Stroke Odysseys study

Journal:	*BMJ Open*
Manuscript ID	bmjopen-2021-057805.R2
Article Type:	Protocol
Date Submitted by the Author:	02-Feb-2022
Complete List of Authors:	Estevao, Carolina; King's College London, nstitute of Psychiatry, Psychology & Neuroscience<br>Baldellou Lopez, Maria ; King's College London Institute of Psychiatry Psychology and Neuroscience, Centre for Implementation Science<br>Davis, Rachel; Florence Nightingale School of Nursing, James Clerk Maxell Building<br>Jarret, Lucinda; Rosetta Life Head Office<br>Soukup, Tayana ; King's College London, Centre Implementation Science<br>Bakolis, Ioannis; King's College London<br>Healey, Andy; King's College London Institute of Psychiatry Psychology and Neuroscience, Centre for Implementation Science; King's College London, King’s Health Economics<br>Harrington, Jean; King’s College London, Department of Psychological Medicine<br>Woods, Anthony; King’s College London, Department of Psychological Medicine<br>Crane, Nikki; King's College London<br>Jones, Fiona; St Georges University of London<br>Pariante, Carmine; King's College London, Psychological Medicine<br>Fancourt, Daisy; University College London Research Department of Epidemiology and Public Health, Department of Behavioural Science and Health<br>Sevdalis, Nick; King's College London
**Primary Subject Heading**:	Qualitative research
Secondary Subject Heading:	Rehabilitation medicine, Research methods, Occupational and environmental medicine, Neurology
Keywords:	Stroke < NEUROLOGY, QUALITATIVE RESEARCH, STATISTICS & RESEARCH METHODS

SCHOLARONE™
Manuscripts

Evaluation of a community-based performance arts programme for people who have experienced stroke in the UK: protocol for the SHAPER-Stroke Odysseys study

Authors

Joint first authors: Carolina Estevao and Maria Baldellou Lopez

Carolina Estevao[1], Maria Baldellou Lopez[2], Rachel Davis[2], Lucinda Jarrett[3], Tayana Soukup[2], Ioannis Bakolis[2,4], Andy Healy[2,5], Jean Harrington[1], Anthony Woods[1], Nikki Crane[6], Fiona Jones[7], Carmine Pariante[1], Daisy Fancourt[8], Nick Sevdalis[2]

[1]Department of Psychological Medicine, Institute of Psychiatry, Psychology and Neuroscience, King's College London, 5 Cutcombe Rd, Brixton, London SE5 9RT, United Kingdom
[2]Centre for Implementation Science, Health Service and Population Research Department, Institute of Psychiatry, Psychology & Neuroscience, 16 De Crespigny Park, Camberwell, London SE5 8AB, United Kingdom
[3]Rosetta Life Head Office, 3 Brook End, Chadlington, Chipping Norton, OX7 3NF, United Kingdom
[4]Department of Biostatistics and Health Informatics, Institute of Psychiatry, Psychology & Neuroscience, 16 De Crespigny Park, Camberwell, London SE5 8AB, United Kingdom
[5]King's Health Economics, Health Service and Population Research Department, Institute of Psychiatry, Psychology & Neuroscience, 16 De Crespigny Park, Camberwell, London SE5 8AB, United Kingdom
[6]Culture team, King's College London, Somerset House East Wing, Strand WC2R 2LS, United Kingdom
[7]Faculty of Health, Social Care and Education, Kingston University and St George's, University of London, London, United Kingdom
[8]Department of Behavioural Science and Health, University College London, Gower Street, London, WC1E 6BT, United Kingdom

Corresponding author: Carolina Estevao[1], carolina.estevao@kcl.ac.uk

Keywords: Performance, stroke, art, community-based, implementation science, dance, poetry, song, music

Word count: 5,120 words

Abstract

Introduction: Stroke survivors, once in the community, face challenges with their long-term rehabilitation care and present higher levels of loneliness, depression, and anxiety than the rest of the population. A community-based performance arts programme, Stroke Odysseys (SO), has been devised to tackle the challenges of living with stroke in the UK. In this study, we aim to evaluate the implementation, impact, and experiences of SO for stroke survivors.

Methods and analysis: SHAPER-SO aims to scale-up Stroke Odysseys to 75 participants and 47 stakeholders, while simultaneously evaluating the effectiveness and implementation of the programme. The main research aim is to evaluate the implementation, effectiveness, impact and experiences of a community-based performance arts programme (SO for stroke survivors). This mixed-methods study will evaluate the experience

For peer review only

and impact of Stroke Odysseys on those participating using mixed methods (interviews, observations and surveys) before and after each stage, and carry out non-participant observations during a percentage of the workshops, training and tour. Data will be analysed using quantitative and qualitative approaches. This is a study within the Scaling-up Health Arts Programmes: Implementation and Effectiveness Research (SHAPER) programme.

Ethics and dissemination: Ethical approval has been granted by the King's College London PNM Research Ethics Panel, REC reference: LRS/DP-20/21-21549. Written informed consent will be sought for participants and stakeholders. The results of the study will be reported and disseminated at international conferences and in peer-reviewed scientific journals.

Study registration: Clinical Trials.gov, NCT04864470.

Strengths and limitations of this study

- The first study examining an art intervention on stroke survivors, using a type 2 hybrid design, with a dual focus on effectiveness and implementation outcomes.
- The unique study design will result in a package of clinical and implementation data on this particular intervention.
- There may be inconsistency in participant experience throughout an intervention period if in-person sessions are switched online and vice-versa due to COVID-19 social distancing restrictions.
- Access to the COVID-19 adapted online delivery of Stroke Odysseys may be challenging for people with severe acquired brain injury resulting from stroke.

Introduction

Stroke affects over 113,000 people every year [1] and, according to the latest statistics, there are currently more than 1.2 million stroke survivors in the UK [2,3]. The effects of stroke are often devastating, with almost two-thirds of survivors leaving the hospital with a disability and half experiencing depression within five years [4,5]. In addition to the substantial impact stroke has on those affected and their caregivers, it can also pose a significant financial burden to health and social care services. The societal cost of stroke has been estimated to be £26 billion per annum, with NHS costs accounting for £3.4 billion in 2015, and projected to increase to £10.2 billion by 2035 [6].

Stroke survivors commonly face emotional, social and psychological challenges, with depression, anxiety and apathy being the most prevalent neuropsychiatric sequelae [7]. Such disabling symptoms are often coupled with feelings of abandonment [8] once hospital rehabilitation ends and their recovery plateaus. Stroke survivors in the UK usually receive rehabilitation whilst in hospital but once they are discharged, the level of support in the community tends to be variable and in the long-term, inadequate for their needs [8]. This is consistent with a meta-review of qualitative systematic reviews [9] which reported a lack of self-management resources available following stroke, highlighting the gap between available services and the long-term social, emotional and physical needs of stroke survivors throughout their rehabilitation journey [10]. Additionally, the findings of a survey by the Stroke Association in the UK emphasised the devastating burden and 'hidden effects' of stroke [11]. The survey, which collated data from over 10,000 stroke survivors and is the biggest to date in the UK, revealed that the effects of stroke on cognition, emotions, relationships, and mental health are widespread, can

For peer review only

be life-long, and are often overlooked or neglected. In the survey, 50% of stroke survivors and 85% of caregivers reported a gap between the support provided versus the support they felt was needed. While current stroke rehabilitation targets functional recovery, it fails to meet the psychosocial needs of stroke survivors.

The evidence summarised above suggests that there is a need for more holistic rehabilitation programmes, especially non-pharmacological and non-invasive modalities, to address the psychosocial needs and improve the quality of life of stroke survivors [12]. Arts-based programmes (such as 'Stroke Odysseys' discussed below) are one such approach that shows promising results in enhancing the wellbeing, self-esteem, social life and rehabilitation experiences of stroke patients [13]. Indeed, over the past decade, several studies conducted in this patient population have consistently shown a positive impact of different art modalities on psychological (e.g. enhancement in confidence and a better sense of control), social (e.g. increased social interactions and peer support) and functional (e.g. improvement in physical abilities) outcomes [12].

Nonetheless, despite the growing body of research on the benefits of art interventions, the process of scaling-up these interventions, embedding them into healthcare and its associated challenges are not yet well-established. Preliminary data indicates that Stroke Odysseys (discussed below) is received positively by those who take part [14], however, identifying barriers to implementation and exploring ways to overcome these obstacles is essential to successfully and sustainably embed SO into clinical pathways and roll out the programme at a wider scale.

Stroke Odysseys is part of the Scaling-up Health-Arts Programme: Implementation and Effectiveness Research (SHAPER), which is, to our knowledge, the world's largest study on arts and health examining both clinical effectiveness and implementation effectiveness of three community-based arts programmes: Melodies for Mums (M4M), a singing intervention for postnatal depression, PD-Ballet, a dance intervention for Parkinson's Disease, and Stroke Odysseys (SO). Overall, SHAPER has three primary aims: (1) to successfully embed each of the art interventions into the healthcare system (i.e. taking a social prescribing approach), (2) to scale up these interventions at a larger scale, and (3) to facilitate these interventions being commissioned by CCGs, ensuring the long-term sustainability of delivery [15].

Stroke Odysseys

Rosetta Life, a well-established non-profit organisation with a track record of conducting arts programmes for stroke and brain injury survivors, developed Stroke Odysseys (SO), a performance-based arts programme with continued consultation from stakeholders (including stroke survivors). SO provides an opportunity for those who have had a stroke or brain injury to share their experiences with an audience through movement, music, songwriting and the spoken word. The programme, which has now been running for over 21 years, uses performance arts to help stroke survivors overcome psychological challenges such as lowered self-esteem, anxiety and depression, which are commonly reported by individuals [16].

In this protocol paper, we present our plans to evaluate. SHAPER-SO will be a two-pronged study, examining the implementation and clinical effectiveness of Stroke Odysseys. The research we will be undertaking examines both, the impact of performance arts on participants and how SO can be embedded into clinical pathways. This will help us to identify not just 'if' but also 'why' the

programme works and support our understanding of how it can be successfully delivered and scaled up within clinical pathways. Alongside this, we will examine participants' experiences of the programme using an ethnographic and constructivist approach. To the best of our knowledge, SHAPER-SO is the first study of its kind in the context of stroke care and rehabilitation.

For peer review only

Aims and objectives

The three main objectives in this study are: (1) to explore the clinical impact (effectiveness) of SO on stroke survivors; (2) to explore SO implementation aspects including uptake, adoption, perceived acceptability, appropriateness, feasibility, the fidelity of receipt, unintended consequences, and sustainability; and (3) to evaluate implementation costs and cost-effectiveness of the intervention, with focus on the costs associated with implementing SO into existing care pathways, health services, partner organisations and commissioning and the impact of scaling up SO on the utilisation of health services.

The main research aim is to evaluate the implementation, effectiveness, impact and experiences of a community-based performance arts programme (SO for stroke survivors).

Our study objectives are as follows:

1. To explore the impact of participation in performance programmes on cognitive health and physical, psychological and social well-being of people that have experienced stroke.
2. To study the context, mechanisms of delivery and interactions between participants and facilitators which take place during SO delivery.
3. To explore the learning and experiences of facilitators and participants after SO delivery.
4. To evaluate any change in the emotional wellbeing, participation, and activity of stroke participants pre- and post-SO
5. To evaluate the extent to which SO is acceptable, feasible to undertake and appropriate to survivors and wider stakeholders (including ambassadors, artists, and clinician referrers to the programme).
6. To, explore the challenges, barriers, facilitators and unintended consequences of the implementation of SO.
7. To assess the costs associated with the implementation of the programme.
8. To, assess the adoption, adherence to it and attrition rates of the programme.

Theoretical underpinning

An ethnographic and constructivist approach will be used to examine stroke survivors' experiences of the SO programme (objective 2). This is described as the study of social interactions, behaviours and perceptions that occurs within groups, team organisation and communities. Ethnography provides rich, holistic insights into people's views and actions, as well as the nature of the location (context) they inhabit. The aim has been described as 'getting inside' the way each group of people sees the world [17]. Ethnography has a strong emphasis on 'unstructured data and involves implicit interpretation of the meaning and function of human interactions, rather than hypothesis testing. This approach aligns well with the complex nature of the Stroke Odysseys programme.

The implementation analyses are informed by several well-established implementation science frameworks, which we have applied to develop a set of implementation facets of SO to assess, both quantitatively and qualitatively (see Methods). We used the recently developed 'Implementation Science Research Development' (ImpRes) framework [17] to identify the elements of implementation that the study ought to capture, ImpRes defined 10 different domains that an implementation evaluation ought to capture – including capturing stakeholder engagement, the outcome of implementation (e.g., how acceptable, appropriate and feasible SO and its implementation processes are to those delivering and also receiving SO) and any unintended consequences (objective 3, 5 and 6). Moreover, we reviewed the Capability, Opportunity, and Motivation Model of Behaviour (COM-B) tool [18] to help us identify any barriers that may affect an individual's engagement with the SO programme (objectives 7 and 10). The COM-B components lie at the centre of the Behaviour Change Wheel (BCW), a framework for designing and characterising behaviour change interventions [18]. The

For peer review only

Consolidated Framework for Implementation Research (CFIR) [19,20] will help us map reported barriers and drivers to the implementation of the SO (objective 7); and finally, the Reach Effectiveness Adoption Implementation Maintenance (RE-AIM) model [21,22] taken together with Proctor et al's [23] taxonomy of implementation outcomes, guided our choice of implementation measures to assess.

Methods and analysis

Design

SHAPER-SO is a mixed-methods programme study, comprising quantitative and qualitative methods to assess the clinical and implementation outcomes outlined in the measures section below.

Intervention

Stroke Odysseys is a post-stroke performance art intervention designed and delivered by the arts organisation Rosetta Life. This intervention initially developed and funded by King's and Guy's & St Thomas' Charity, aims to improve recovery, agency and well-being after stroke [14].

Stroke Odysseys comprises three distinct stages 1) weekly workshops conducted over 12 weeks for stroke participants which will be facilitated by an integrated team of expert artists and 'stroke ambassadors' from the charity Rosetta Life, 2) a smaller group of ambassadors recruited from the workshops will be trained to become co-facilitators (i.e. new stroke ambassadors), 3) a performance tour including education and taster workshops for audiences.

During sessions, which run for three hours each, participants devise a dance and music performance work from their own stories. The practice of "performing ourselves" is key to achieving successful outcomes such as transforming the participants' perception of identity. The culmination of the programme will be a public-facing performance to an audience of carers, health care practitioners, friends, family and the wider community.

Due to the ongoing COVID-19 pandemic and the necessity of shielding vulnerable adults and foreseeing increased anxiety in stroke survivors to attend in-person sessions, we have adapted the SO programme to be delivered through a mixture of live/face-to-face and online delivery (blended approach). Participants will be able to choose whether to attend the sessions/participate face-to-face or online based on their personal preferences and needs. The researcher will manage groups to ensure that all the participants that wish to attend in person will be able to do so during the 12-weeks.

The adapted programme will still be run in three stages, described below:

Stage 1) The workshops are the result of co-creation; the general framework is: weeks 1 - 3 building the performance company, weeks 4 - 6 devising the performance weeks 6 - 9 rehearsing the performance and weeks 10 - 12 are sometimes concertina-ed into one production week introducing stage management, lighting and technical runs. Each of the 12 workshops contains a performance "class" of 20 - 30mins exploring movement and voice techniques and exercise.

Participants will be able to choose whether to attend the sessions/participate face-to-face or online based on their personal preferences and needs.

Stage 2) After the performance is completed, participants will be invited to a four-day training programme where they will learn to act as advocates for life after stroke – termed 'stroke ambassadors'. The optional ambassador training starts with an introduction to being an ambassador and an outline of the pathways available: a) supporting artists in hospital and community contexts, b) speaking the press and media / advocating for life after stroke, c) engaging in academic research and d) joining the steering group that informs activities and directions. The skills development training is delivered in three stages: an introduction to movement practices and the traditions of independent dance, then an introduction to voice and improvisation and finally, an introduction to performance. Each ambassador then constructs an individually tailored programme according to their personal goals and intentions in becoming an ambassador.

For peer review only - http://bmjopen.bmj.com/site/about/guidelines.xhtml

The programme will take place once weekly and will be led by a team of artists and supported by a leadership coach. All training will take place on Zoom until social distancing measures are lifted, and participants are willing to meet indoors – a blended ambassador training will be offered.

Stage 3) Following training, a volunteer manager will coordinate a tailored programme where ambassadors support artists in recruitment, befriend the newly discharged stroke survivors and take part in small scale performance tours to challenge the perception of disability. The tour will be delivered online with online screenings followed by Q&A with ambassadors, taster sessions and exercises delivered online with the ambassadors.

The programme will be delivered in two cycles of the complete 3-stage intervention. At the end of the two cycles of the programme, a group of newly trained ambassadors will emerge. The programme seeks to develop a national network of Ambassadors who will build capacity for performance arts in healthcare and a wider capacity for healthcare. The Stroke Ambassadors are graduates of the 12-week workshop that receive training, based on a leadership-coaching model, and they deliver a tailored advocacy programme according to their creative skills – befriending, performance administration and support, programme advocacy.

Study setting

The study will take place online until conditions of the pandemic enable researchers, artists, and participants to meet safely indoors, as per government guidelines. When it is feasible and safe to meet in person, participants, artists, and researchers will meet in an established performance arts education centre to ensure that COVID guidelines on cleanliness are guaranteed. When ran in person, the workshops are run in Central London locations, with a single centre running the programme in each cycle.

Sample and recruitment

Stroke survivors

Consenting stroke participants will be included if they are:

(1) over 18 years of age
(2) have had one or more stroke(s)
(3) received inpatient care in a UK stroke care pathway
(4) able to follow a 2-stage command and hold a conversation in English if no supporter/friend is available to translate

The following exclusion criteria will be applied to individuals:

(1) with co-morbidities that would prevent participation in group activities (e.g. dementia or deteriorating or fluctuating palliative conditions)
(2) unable to understand English
(3) unable to commit to the 12-week programme

Additionally, stroke ambassadors will be included if they have been through the ambassador training and are involved in at least one programme cycle culminating in the tour.

All participants will be offered the option of completing an interview with their carer present. This will be offered both after the first 12-week programme and after the ambassador training, for those that wish to participate. Those that decline will be asked if they would be willing to provide their reasons why.

Wider stakeholder group

For peer review only

In addition to the stroke survivors that enrol on the SO programme, data will also be collected from a wider stakeholder group involved in the delivery or support of the programme. Individuals will be recruited if they meet the following criteria:

(1) over 18 years of age
(2) can hold a conversation in English if no supporter/friend is available to translate
(3) can either be defined as a:
  - Supporters: family members or carers.
  - Deliverers: individuals responsible for the delivery of the research (facilitators and artists).
  - Referrers: individuals involved in signposting (e.g. doctors, nurses, healthcare workers).

Wider stakeholders will be excluded from participation if they are unable to understand English or if no supporter/friend is available to translate.

Sampling

Sample size

We aim to recruit 75 new stroke survivors in total for the duration of the study. A prediction of 75 participants has been estimated based on the numbers that over the years running Stroke Odysseys, *Rosetta Life* has been able to recruit in two consecutive cycles. This number has also considered the organisation being able to whilst maintain a manageable ratio of participants to artists and staff members, guaranteeing that Stroke Odysseys is delivered to the highest standard.

Based on previous experience of running SO where participants then complete an ambassador training cycle, a drop-out rate of 20% is expected and so the final number of ambassadors that complete the ambassador training is estimated to be 60.

The wider stakeholder group will be recruited from the network of people who are involved in the programme and present in the community. This includes the voluntary sector, health and social care sectors and clinical commissioners. A total of 47 stakeholders, a forecast based on the existing network numbers, will be recruited (12 carers, 10 clinical team members, 5 artists, 20 existing ambassadors).

Recruitment procedure

Potential stroke survivor participants will be identified through signposting in community centres and care homes as well as engaging in presentations, screenings, taster sessions and performances during the tour.

Recruitment of potential participants will be done online. Screenings of performance extracts will be followed by taster sessions online and a Q&A with ambassadors. Potential participants will be directed to the project manager at Rosetta Life who will manage all referrals.

Potential participants will be offered a PIS and an ICF and will be explained the details of the study. Written consent will be sought following a 48h colling-off period.

Wider stakeholders will be recruited from the networks of people involved in the referral, delivery or support of the programme.

A recruitment log will be kept by the research team to accurately record included and excluded participants as well as missing data from dropouts to account for possible sampling bias.

Study flowchart

A study overview can be seen in the flowchart below (Figure 1):

Data collection

For peer review only - http://bmjopen.bmj.com/site/about/guidelines.xhtml

This is a prospective mixed-methods study using a range of qualitative and quantitative methods at different time points pre-, during and post-intervention of each programme cycle.

Qualitative methods will comprise semi-structured interviews and non-participant observations of training and production to assess experiences and attitudes towards the programme and its implementation.

Quantitative methods will be used to assess experiences and attitudes towards the SO programme and its implementation. Further information is included further in the 'methods' section in the outcome measures tables (Tables 1 and 2) and the 'assessment descriptions' section.

Demographic data will be collected by Rosetta Life at the time of enrolment.

Outcomes

Data on the clinical outcomes will be collected from stroke survivors who have enrolled on the SO programme (see Table 1). Data on the implementation outcomes will be collected from stroke survivors who have enrolled on the SO programme as well as the wider stakeholder group involved in the SO programme, including deliverers, referrers, and supporters (see Table 2).

For peer review only

Table 1. Clinical outcomes

Objective	Clinical Outcome Measures/Endpoints	Type of assessment	The time point for data collection
**Primary objective**			
**Secondary objectives**			
To study the context, mechanisms and interactions which take place during Stroke Odysseys delivery	Non-participant observations of workshops	Qualitative	T1 (during workshop delivery)
To explore the learning and experiences of facilitators and participants	Semi-structured interviews- stroke participants and facilitators	Qualitative	T2
To explore stroke survivors' preparation and participation in performances	Semi-structured interviews- stroke participants	Qualitative	T0

Note: Data on the clinical outcomes will be collected from stroke survivors who have enrolled on the SO programme

Table 2. Implementation outcomes

Objective	Implementation Outcome Measures/Endpoints	Type of assessment	Time points for data collection	Who data will be collected from
Primary objective				
To evaluate to what extent Stroke Odysseys is acceptable, to survivors and wider stakeholders	Acceptability of intervention Measure (AIM)<br><br>Semi-structured interviews (to explore reasons for acceptability score)	Quantitative<br><br>Qualitative	T1, T2, T3<br><br>T2, T3	Stroke survivors, deliverers, supporters, referrers<br><br>Stroke survivors, deliverers, supporters, referrers
Secondary objectives				
To evaluate to what extent Stroke Odysseys are appropriate to survivors and wider stakeholders	Intervention Appropriateness Measure (IAM)<br><br>Semi-structured interviews (to explore reasons for appropriateness score)	Quantitative<br><br>Qualitative	T1, T2, T3<br><br>T2, T3	Stroke survivors, deliverers, supporters, referrers<br><br>Stroke survivors, deliverers, supporters, referrers

For peer review only

To evaluate to what extent Stroke Odysseys feasible to survivors and wider stakeholders	Feasibility Intervention Measure (FIM)<br>Semi-structured interviews (to explore reasons for feasibility score)	Quantitative<br><br>Qualitative	T1, T2, T3<br><br>T2, T3	Stroke survivors, deliverers, supporters, referrers<br><br>Stroke survivors, deliverers, supporters, referrers
To assess any unintended consequences of the programme	Semi-structured interviews	Qualitative	T2, T3	Stroke survivors, deliverers, supporters, referrers
To explore the facilitators and barriers to implementing the programme	Semi-structured interviews	Qualitative	T2, T3	Stroke survivors, deliverers, supporters, referrers
To explore the facilitators and barriers to sustained use of the programme	Semi-structured interviews	Qualitative	T2, T3	Stroke survivors, deliverers, supporters, referrers
To assess service utilisation and cost associated costs and changes in quality of life associated with the implementation of the programme	EQ5D-5L (quality of life measure) and AD-SUS (adult service receipt schedule) and semi-structured interviews and activity data (to estimate implementation costs).	Quantitative	T2 and T3	Stroke survivors<br><br>Stroke survivors, deliverers, supporters, referrers
To explore the strategies including resource inputs utilised, used within individual sites to implement the programme	Semi-structured interviews	Qualitative	T2, T3	Deliverers, referrers
To assess the adoption of the programme	The number of individuals delivering the programme, and the number of individuals supporting the programme (and continuing to do so)	Quantitative	T0, T2, T3	Deliverers, referrers
To assess programme adherence and attrition rates	Data on the overall adherence to the programme, number of drops-outs and reasons why	Quantitative<br><br>Qualitative	Data recorded from the register on weekly attendance rates for the 12-week programme (stage 1) and 4-week ambassador programme (stage 2)<br><br>T2, T3	Deliverers (record data)<br><br>Stroke survivors

Notes: Data on the implementation outcomes will be collected from stroke survivors who have enrolled on the SO programme as well as the wider stakeholder group involved in the SO programme (including deliverers, referrers and supports).

For peer review only

Timepoints for data collection: T0 – Baseline; T1- Midway through the 12-week programme (weeks 5-7); T2 - Immediately post-performance (12-14 weeks); T3 - Immediately after the advocacy training for stroke ambassadors.

For peer review only

To maximise inclusivity and outcome completion, and minimise participant burden, outcome assessments, where possible, will be conducted either face-to-face, online, by telephone or via postal questionnaire depending on the outcome measures being assessed, participants preferences and government COVID-19 guidelines.

Assessment descriptions for clinical outcomes

Qualitative assessments

Ethnographic research

Ethnographic non-participant observations of a selection of the 12 workshops including at least 1-2 groups from each of the two phases (building confidence, rehearsal and production) to capture facilitator and participant practice, interactions and routines. Each observation period will last for the duration of the workshop, and the ethnographic researcher will record field notes contemporaneously.

Semi-structured interviews

Semi-structured interviews will be held with facilitators and participants pre- and post-programme cycles to explore anticipated concerns and expectations (pre) and experiences of facilitation and factors influencing delivery, engagement of participants, adaptation, and learning (post).
The implementation science research team will be interviewing participants across both, the 12-week programme and ambassador training, in addition to wider stakeholders.

Assessment descriptions for the implementation outcomes

Quantitative assessments

Validated and standardised implementation scales will be used to gather quantitative data on how acceptable, appropriate, and feasible the SO programme is perceived by stroke survivors, ambassadors, deliverers, supporters and referrers. These scales include the Acceptability of Programme Measure (AIM), the Programme Appropriateness Measure (IAM) and the Feasibility of Programme Measure (FIM. For further information on the development of these scales, please refer to the paper by Weiner et al [24].
The implementation science researchers will quantify and cost the resources used in implementing the programme, evaluate wider service utilisation and associated costs before and after participants complete the programme, including any changes to their quality-of-life profile measured using the EQ5D-3L preference-based QoL measure. The EQ5D-3L is a self-complete multi-attribute measure of health-related quality of life that assigns individuals a unique state of health based on their response to individual items. Each unique health state is associated with a pre-determined "utility" value derived from a survey of wider community preferences over different states of health. The utility-scale is anchored at 1 (full health) and zero (death), with negative values allowed in instances where states of health are considered worse than death. Health state utility values are subsequently used to estimate quality-adjusted years survived over time (QALYs) – the utility scores providing the means of making the quality adjustments. Evidence on costs and QALYs will subsequently be used to inform an analysis of the cost-effectiveness of programme delivery at scale.

Qualitative data collection

Semi-structured interviews

For peer review only

Semi-structured interviews will be conducted with a purposive sub-sample of stroke survivors (N= 20: 5 from each cycle at two-time points – T2 and T3). Interviews will be carried out with this sub-sample of stroke survivors to explore their attitudes towards the acceptability, appropriateness, and feasibility of the programme, as well as factors (facilitators or barriers) that affected their involvement (and potential drop-out) and any unintended consequences. These issues will also be explored with a sub-sample of individuals (10 in total) from each of the wider stakeholder groups.

Interview guides have been based on existing implementation frameworks (see above) and adapted from a previous project [25]. They will be further adapted and co-designed with our stakeholder group to ensure the questions in the interview guide are meaningful and address the core aims of the study.

Interviews will be audiotaped and are anticipated to be conducted 1:1 or in participants dyads, face to face (government guidelines permitting) or remotely by phone or video.

Data analysis

Data will be analysed using quantitative and qualitative approaches.

Quantitative analysis

Descriptive statistics of survey data will be performed (frequency distribution, central tendency). Parametric and non-parametric tests will also be employed to compare the survey responses to the AIM, FIM, IAM and EQ5D before and after the SO intervention. Changes in AIM, FIM, IAM and EQ5D will be assessed using generalised linear models depending on the distribution of the outcome (continuous, binary, ordinal). All analyses will be conducted in STATA V.14.1.

Qualitative analysis

Initial analysis of qualitative data will be undertaken using an inductive approach to thematic analysis. All data from interviews and observations will be managed using NVivo 10 and examined to categorise themes and key issues that emerge. Using this inductive approach, tentative theoretical explanations will be generated for each sub-group. Summary memos for data sets will be developed for each sub-group to provide the basis for within and between-group comparisons. The inductive approach is data-driven; based on observation, the early analysis seeks to reveal patterns and themes from which tentative hypothesis can be drawn subsequently leading to theory; theories are devised to explain what is seen rather than the other way around.

CFIR (www.CFIR.org) will be used to further guide the coding and analysis (i.e. framework analysis) of interview data to identify barriers and facilitators to the implementation and sustainment of the SO programme. This approach has been used previously, that is, CFIR has been applied post-implementation to investigate facilitators and barriers to implementation among stakeholders who had already adopted and implemented an innovation, thus identifying determinants of implementation posthoc [26,27].

Reflective summaries: The relationship of the researcher(s) with the research context they are investigating will be presented in the form of a written narrative of ideas and experiences during data collection. These reflective summaries will be shared with the research team and externally to judge any possible biases with the way the data was collected or prior assumptions.

For peer review only

Patient and public involvement

The programme has been developed and further refined using co-design methodologies with a group of 20 members of South London stroke communities. The project has been shared widely with stroke clinicians across London and has their full support. During the pandemic Rosetta Life set up an advisory group consisting of Stroke Ambassadors to support the redesign of the website www.strokeodysseys.org, to monitor how people living with the effects of a stroke were engaging with the online workshops, to oversee the development of the education videos and the Ambassadors Handbook.

This advisory group is now a stable and national network of Ambassadors who curate an online programme and advise on the development and delivery of SO. They have advised the investigators on the need to ensure that the measures were aphasia friendly and found an organisation to make sure that the measures were aphasia friendly. They will now look at the language of the Implementation Science measures and make sure that they are accessible.

Trial registration and current status

This study is registered on ClinicalTrials.gov PRS under the ClinicalTrials.gov ID: NCT04864470. Recruitment was scheduled to start in Autumn 2021.

Data protection

The Investigator will ensure that this study is conducted in full conformity with relevant regulations and with the ICH Guidelines for Good Clinical Practice (CPMP/ICH/135/95) July 1996. The Investigator will ensure that this study is conducted in accordance with the principles of the Declaration of Helsinki.

Access to person identifiable implementation science data will rest with the data custodian(s) from the immediate study team and the implementation science team. Since the project seeks to explore in some depth participants' experiences and barriers and facilitators to implementation, it is important to maintain strict confidentiality and facilitate openness in the interviews and survey responses thus optimal data quality.

Consent forms and audio/video recordings will be kept electronically in KCL's SharePoint for the duration of the study, only accessible by the teams at KCL, Kingston University and Rosetta Life involved in the study. Consent forms and other identifiable paperwork will be kept in locked cabinets only accessible to the study team. Study data will be kept in a separate location from the person identifiable information. Access to the de-identified research data will be shared with the study management group for the purposes of review, analysis and dissemination. Only de-identified data will be analysed.

After the completion of the study, the study data will be kept for the King's College London's standard retention period of 10 years after the completion of the study. The study data that supports published results will be deposited in a secure data repository (e.g. King's Research Data Management System). This will allow the data to be accessible for future reuse as per King's College London's policy on the management of research data long-term.

Ethics and dissemination

For peer review only

Ethical approval has been granted by the King's College London PNM Research Ethics Panel, REC reference: LRS/DP-20/21-21549. Informed consent will be collected in writing from all research participants and stakeholders involved in the study. Findings will be published in peer-reviewed journals and disseminated at national and international meetings.

References

1 Rothwell PM, Coull AJ, Giles MF, *et al.* Change in stroke incidence, mortality, case-fatality, severity, and risk factors in Oxfordshire, UK from 1981 to 2004 (Oxford Vascular Study). *Lancet* 2004;**363**:1925–33. doi:10.1016/S0140-6736(04)16405-2

2 National Institute for Health and Care Excellence. NICE impact stroke. 2019. https://www.nice.org.uk/Media/Default/About/what-we-do/Into-practice/measuring-uptake/NICE-Impact-stroke.pdf (accessed 23 Jul 2021).

3 Stroke Association. Stroke statistics. Stroke Association. 2020.https://www.stroke.org.uk/what-is-stroke/stroke-statistics (accessed 23 Jul 2021).

4 Adamson J, Beswick A, Ebrahim S. Is stroke the most common cause of disability? *J Stroke Cerebrovasc Dis* 2004;**13**:171–7. doi:10.1016/j.jstrokecerebrovasdis.2004.06.003

5 Ayerbe L, Ayis S, Crichton S, *et al.* The long-term outcomes of depression up to 10 years after stroke; the South London Stroke Register. *J Neurol Neurosurg Psychiatry* 2014;**85**:514–21. doi:10.1136/jnnp-2013-306448

6 King D, Wittenberg R, Patel A, *et al.* The future incidence, prevalence and costs of stroke in the UK. *Age and Ageing* 2020;**49**:277–82. doi:10.1093/ageing/afz163

7 Ferro JM, Caeiro L, Figueira ML. Neuropsychiatric sequelae of stroke. *Nat Rev Neurol* 2016;**12**:269–80. doi:10.1038/nrneurol.2016.46

8 Pindus DM, Mullis R, Lim L, *et al.* Stroke survivors' and informal caregivers' experiences of primary care and community healthcare services - A systematic review and meta-ethnography. *PLoS One* 2018;**13**:e0192533. doi:10.1371/journal.pone.0192533

9 Pearce G, Pinnock H, Epiphaniou E, *et al.* Experiences of Self-Management Support Following a Stroke: A Meta-Review of Qualitative Systematic Reviews. *PLOS ONE* 2015;**10**:e0141803. doi:10.1371/journal.pone.0141803

10 Roads to Recovery - Organisation and quality of stroke services. NIHR Evidence. doi:10.3310/themedreview-001685

11 Lived Experience of Stroke. Stroke Association 2019. http://www.stroke.org.uk/sites/default/files/conferences/nisc/documents/lived_experience_of_stroke_chapter_1.pdf

12 Lo TLT, Lee JLC, Ho RTH. Creative Arts-Based Therapies for Stroke Survivors: A Qualitative Systematic Review. *Front Psychol* 2018;**9**:1646. doi:10.3389/fpsyg.2018.01646

13 Chan CKP, Lo TLT, Wan AHY, *et al.* A randomised controlled trial of an expressive arts-based intervention for young stroke survivors. *BMC Complementary Medicine and Therapies* 2021;**21**:7. doi:10.1186/s12906-020-03161-6

For peer review only

14 Jarrett L. Stroke Odysseys: performance arts after stroke, a service innovation for neurological rehabilitation. *British Journal of Neuroscience Nursing* 2018;**14**:S18–21. doi:10.12968/bjnn.2018.14.Sup5.S18

15 Estevao C, Fancourt D, Dazzan P, *et al.* Scaling-up Health-Arts Programmes: the largest study in the world bringing arts-based mental health interventions into a national health service. *BJPsych Bulletin* 2021;**45**:32–9. doi:10.1192/bjb.2020.122

16 Barker-Collo SL. Depression and anxiety 3 months post-stroke: prevalence and correlates. *Arch Clin Neuropsychol* 2007;**22**:519–31. doi:10.1016/j.acn.2007.03.002

17 Hull L, Goulding L, Khadjesari Z, *et al.* Designing high-quality implementation research: development, application, feasibility and preliminary evaluation of the implementation science research development (ImpRes) tool and guide. *Implementation Science* 2019;**14**:80. doi:10.1186/s13012-019-0897-z

18 Michie S, van Stralen MM, West R. The behaviour change wheel: A new method for characterising and designing behaviour change interventions. *Implementation Science* 2011;**6**:42. doi:10.1186/1748-5908-6-42

19 The Consolidated Framework for Implementation Research. https://cfirguide.org/tools/ (accessed 21 Sep 2021).

20 Damschroder LJ, Aron DC, Keith RE, *et al.* Fostering implementation of health services research findings into practice: a consolidated framework for advancing implementation science. *Implementation Science* 2009;**4**:50. doi:10.1186/1748-5908-4-50

21 RE-AIM – Reach Effectiveness Adoption Implementation Maintenance. https://www.re-aim.org/ (accessed 21 Sep 2021).

22 Glasgow RE, Vogt TM, Boles SM. Evaluating the public health impact of health promotion interventions: the RE-AIM framework. *Am J Public Health* 1999;**89**:1322–7. doi:10.2105/ajph.89.9.1322

23 Proctor E, Silmere H, Raghavan R, *et al.* Outcomes for Implementation Research: Conceptual Distinctions, Measurement Challenges, and Research Agenda. *Adm Policy Ment Health* 2011;**38**:65–76. doi:10.1007/s10488-010-0319-7

24 Weiner BJ, Lewis CC, Stanick C, *et al.* Psychometric assessment of three newly developed implementation outcome measures. *Implementation Science* 2017;**12**:108. doi:10.1186/s13012-017-0635-3

25 Soukup T, Hull L, Smith EL, *et al.* Effectiveness-implementation hybrid type 2 trial evaluating two psychoeducational programmes for severe hypoglycaemia in type 1 diabetes: implementation study protocol. *BMJ Open* 2019;**9**:e030370. doi:10.1136/bmjopen-2019-030370

26 Kirk MA, Kelley C, Yankey N, *et al.* A systematic review of the use of the Consolidated Framework for Implementation Research. *Implementation Science* 2016;**11**:72. doi:10.1186/s13012-016-0437-z

27 Proctor E, Silmere H, Raghavan R, *et al.* Outcomes for Implementation Research: Conceptual Distinctions, Measurement Challenges, and Research Agenda. *Adm Policy Ment Health* 2011;**38**:65–76. doi:10.1007/s10488-010-0319-7

For peer review only

Figure legends:
Figure 1: SHAPER-SO study flowchart

Contributors

All authors listed have contributed to the conception and design of the protocol and this manuscript. All authors have been involved in the drafting of the manuscript and have individually approved the version of the work published. Specifically, the contribution of each author falls within the following CRediT categories: CE, MBL, RD, TS, IB, JH, AH, FJ, DF and NS: conceptualization, methodology and project administration. AW, NC, CP: conceptualisation, project administration and funding acquisition.
LJ: conceptualisation, project administration.

Funding

This trial is part of the SHAPER programme, a Scaling-up Health-Arts Programme to scale up arts programmes. This programme is funded by the Wellcome Trust (award reference 219425/Z/19/Z). This work is additionally supported by the National Institute for Health Research (NIHR) Biomedical Research Centre at South London and Maudsley NHS Foundation Trust and King's College London and by an NIHR Senior Investigator to CMP.

Competing interests

NS is the director of London Safety and Training Solutions Ltd, which offers training in patient safety, implementation solutions and human factors to healthcare organisations and the pharmaceutical industry. CMP reports grants from the Wellcome Trust, during the conduct of the study; and grants from the National Institute for Health Research (NIHR), NIHR Senior Investigator, Johnson & Johnson, and the Wellcome Trust, outside the submitted work. The other authors have no conflicts of interest to declare.

For peer review only

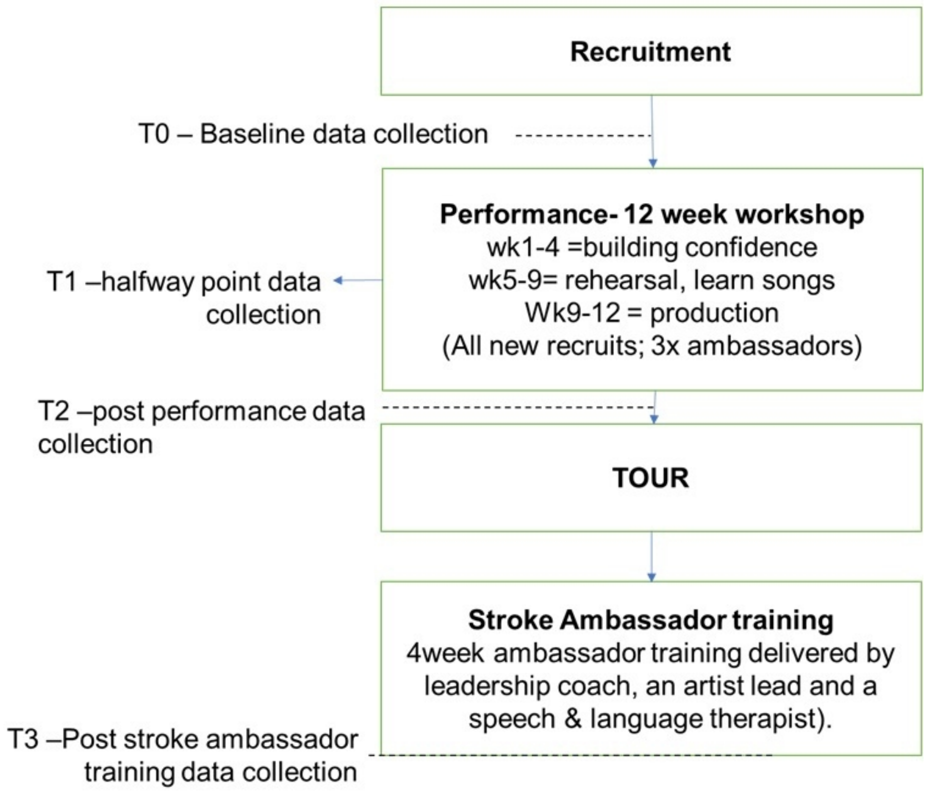

SHAPER-SO flowchart

144x119mm (144 x 144 DPI)
